# Supplementary material for: Slab Grave expansion disrupted long co-existence of distinct Bronze Age herders in central Mongolia
Source: Nat Commun. 2025 Sep 25;16:8420. doi: 10.1038/s41467-025-63789-1 (PMC12462455; doi:10.1038/s41467-025-63789-1)
Supplement: Supplementary file 1 — Supplementary Information [file 41467_2025_63789_MOESM1_ESM.pdf]

Supplementary Information for

Slab Grave expansion disrupted long co-existence of distinct Bronze Age herders in central Mongolia

Juhyeon Lee[†], Ursula Brosseder[†,*], Hyungmin Moon, Raphaela Stahl, Lena Semerau, Jamiyan-Ombo Gantulga, Jérôme Magail, Jan Bemmann, Christina Warinner*, Choongwon Jeong*

[†] These authors contributed equally

* Corresponding author. Email: cwjeong@snu.ac.kr (C.J.), warinner@fas.harvard.edu (C.W.), ursula.brosseder@leiza.de (U.B.)

This PDF file includes:

Supplementary Notes 1 and 2

Supplementary Figures 1 to 16

Other Supplementary Materials for this manuscript include the following:

Supplementary Data 1 to 18

Supplementary Note 1. Archaeological Comment on grave forms and cultural affiliation

Ursula Brosseder

Typology of graves

The typology for grave forms has been developed within our research project "Bioarchaeological research on cemeteries in the Upper Orkhon Valley (BARCOR)" at the site Maikhan Tolgoi. For the typology and chronology at the site see also [1].

Sagsai type graves: These graves are mostly square, rarely round in shape, have a frame made of larger rocks and a fill of several layers of stones. The center of these structures is either flat or shows a small central mound. Almost perfectly aligned with the cardinal directions (north, east, south and west) are corner constructions of larger stones propped against each other or single large standing stones. Removing the fill reveals a central mound made of larger rocks that covered a central pit. The deceased were buried with the head oriented north to north-northwest in supine position or on their side.

Mounds: They are constructed like Sagsai type burials, but without stone constructions marking the cardinal directions or corners. A frame of large boulders, a fill several layers high, cover a central mound built on top of a burial pit. The deceased were buried in supine position with the head oriented towards north-northwest. Future research will show how this large group of graves may be further divided.

Platform mound (Maikhan Tolgoi no. 21): Unique round burial measuring 20.6 m in diameter. The frame is made of large boulders with a fill that consists of two layers: large rocks on the bottom were covered by smaller rocks to achieve the platform-like appearance. After removing the fill, a central mound was uncovered. Below, a very narrow burial pit with a deceased placed on the side with its head oriented to the north-northwest.

Khirgisuur: Typical for this phase are khirgisuurs. Those are central stone mounds which cover a pit or a stone cist. In Maikhan Tolgoi itself, the orientation of the skeleton in supine position is north-northwest. The surrounding area between the central mound and to the either round or square fence can be filled with one layer of densely or loosely set small stones. Sometimes a ring surrounds the central burial mound. Two types of accompanying structures are characteristic: a) ring-shaped stone settings – so-called satellites – frame a pit with burnt caprine or bovine bones, b) stone mounds cover head and hooves of a horse. The satellites can occur on different sides, however the horse head mounds are mostly found on the southeastern side in Maikhan Tolgoi.

Figure-shaped graves: Rectangular burials with their long sides bent inward, giving the structure an hour-glass appearance. The construction is framed by larger, often upright standing stone slabs. Several stone layers make up the fill. The deceased is buried in a pit, often located off-center with their head in southeastern direction. Based on one burial excavated in Saikhan Bulagiin Denzh and in comparison with the literature, the deceased were buried in prone position.

Dumbbell-shaped graves: Those are a variation of figure-shaped burials with upright standing slabs constituting the frame, but have extremely rectangular shaped short ends that give the overall structure the shape of a dumbbell. The deceased were buried with their heads southeast in prone position.

Small mounds with nested circular construction: Small mounds that have a nested frame construction and a deceased buried in supine position with its head in southeastern direction in a burial pit.

The following grave types have not been identified in Maikhan Tolgoi but are mentioned in the literature, and a short description is provided.

Ulaanzuukh type graves: Mostly rectangular graves with straight sides. The construction varies, some have an enclosure, and the fill can vary with, sometimes only one layer of stones. The deceased is buried with its head in southeastern direction and in prone position [2].

Multi-animal offering burials (MAOB): Multi-animal offering burials (MAOB) are mostly rectangular enclosures, sometimes with a stones piling up around it, with a deep burial pit and numerous animal offerings on a ledge in the upper part of the pit. The deceased is oriented east and buried in supine position [3].

Comment on Cultural Affiliation

This is not the place to solve the problem of defining an archaeological culture nor narrate the history of the various attempts of classification, thus the following is a comment on the current state of research. Currently, archaeologists are working towards a more comprehensive and detailed understanding of the meaning of different grave forms within these two large cultural phenomena that we name here DSKC (west/central) and figure-shaped (south/east).

The DSKC (Deer Stone Khirgisuurs Complex) is rather well defined. Work by Frohlich and colleagues [5,6] made a first comprehensive attempt in English to group the mounds, khirgisuurs and deer stones [7]. Houle [8] made similar observations regarding the different mounds. More recent research in western Mongolia, especially by Ts. Turbat and his team have led to the definition of Sagsai mounds [9-11]. They could also show the continuity and temporal sequence from Sagsai to khirgisuurs as both use the same cemeteries in western Mongolia [12,13] as well as in central Mongolia [1,14], supporting their grouping as DSKC.

The burials in the east and south of Mongolia have been grouped differently and several names have been suggested. The different approaches of categorizing emphasize different aspects of the burials (grave layout, construction, position of the deceased, especially prone positioning). Details of the typology have not yet been worked out, also because for some burials there are only a few specimens known [1] and different suggestions exist [15]. Erdenebaatar [16] proposed the term “Tevsh culture” emphasizing less the form but the construction type using vertical slabs and the prone burial position. Honeychurch combines the graves of the Ulaanzuukh type and figure-shaped graves under the umbrella term of “Ulaanzuukh-Tevsh culture” [17], Miyamoto named it “Stone-slab burial culture” [18], Amartuvshin suggested the term “Prone Position Culture” [2]. Despite the ongoing research working out details of naming this eastern group and the typology/chronology of grave types, a consensus exists among the archaeologists that the graves found in southern and eastern Mongolia are as a group following a different set of rules that unites them and sets them distinctively apart from the DSKC archaeological culture. For our paper we chose “figure-shaped” to designate their cultural affiliation.

Supplementary Note 2. Bayesian Modeling for the overlap of Khirgisuurs and Slab burials in the Orkhon Valley

Ursula Brosseder

Based on a relatively small dataset of 24 graves, Bayesian modeling suggests an overlap of approximately 150 years between the use of khirgisuur monuments and the emergence of Slab Grave burials in the Orkhon Valley. The model estimates that khirgisuur construction ended between 1021 and 873 BCE (Supplementary Fig. 6), while Slab Grave construction began between 1237 and 1032 BCE (95% confidence interval) (Supplementary Fig. 7), yielding a median overlap of 157 years (Supplementary Fig. 5). This overlap is estimated to span from around 1100 to 950 cal BCE, with median values refining this range to 1116–965 cal BCE (Supplementary Fig. 5). Although the precision of these start and end dates would benefit from a larger dataset, the evidence clearly excludes the possibility of no overlap between these mortuary practices.

Acknowledgement: I thank Dr. Richard Staff, School of Archaeology, University of Oxford discussing the dates with me and helping with the images Supplementary Figs. 5, 6 and 7.

Supplementary Figures

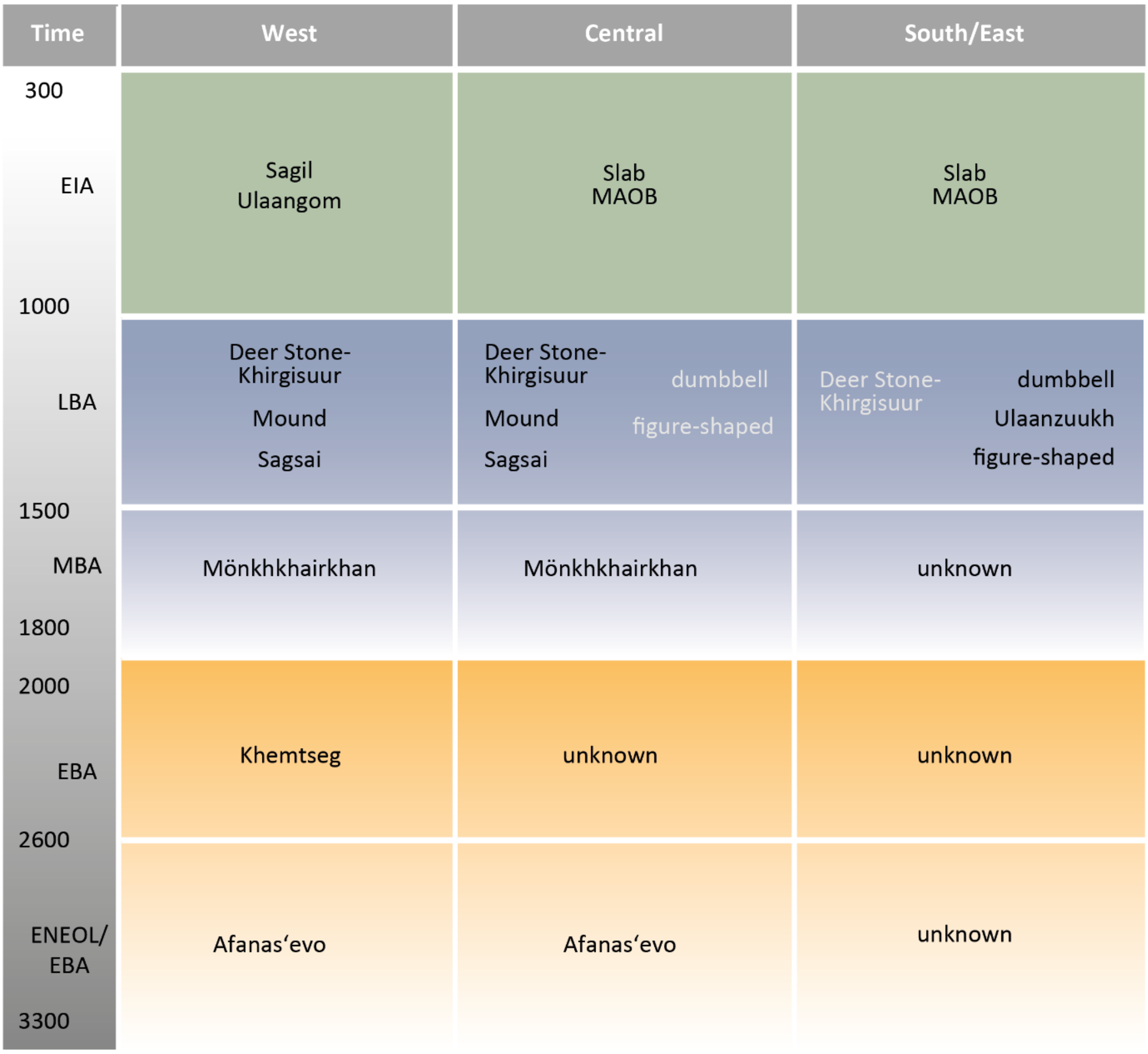

Supplementary Figure 1. Chronology and burial traditions in Mongolia. The chronological framework and associated burial traditions in Mongolia are shown in the figure. Time (in BCE) is depicted along the left side, while the temporal distribution of grave types and their geographical occurrence are shown across three regions: western, central, and south/eastern Mongolia. Changes in background color signify major mortuary transitions. Grave forms written in black represent dominant occurrences, whereas those in white indicate minor occurrences in the respective area. MAOB stands for multi-animal offering burials, described in Supplementary Note 1.

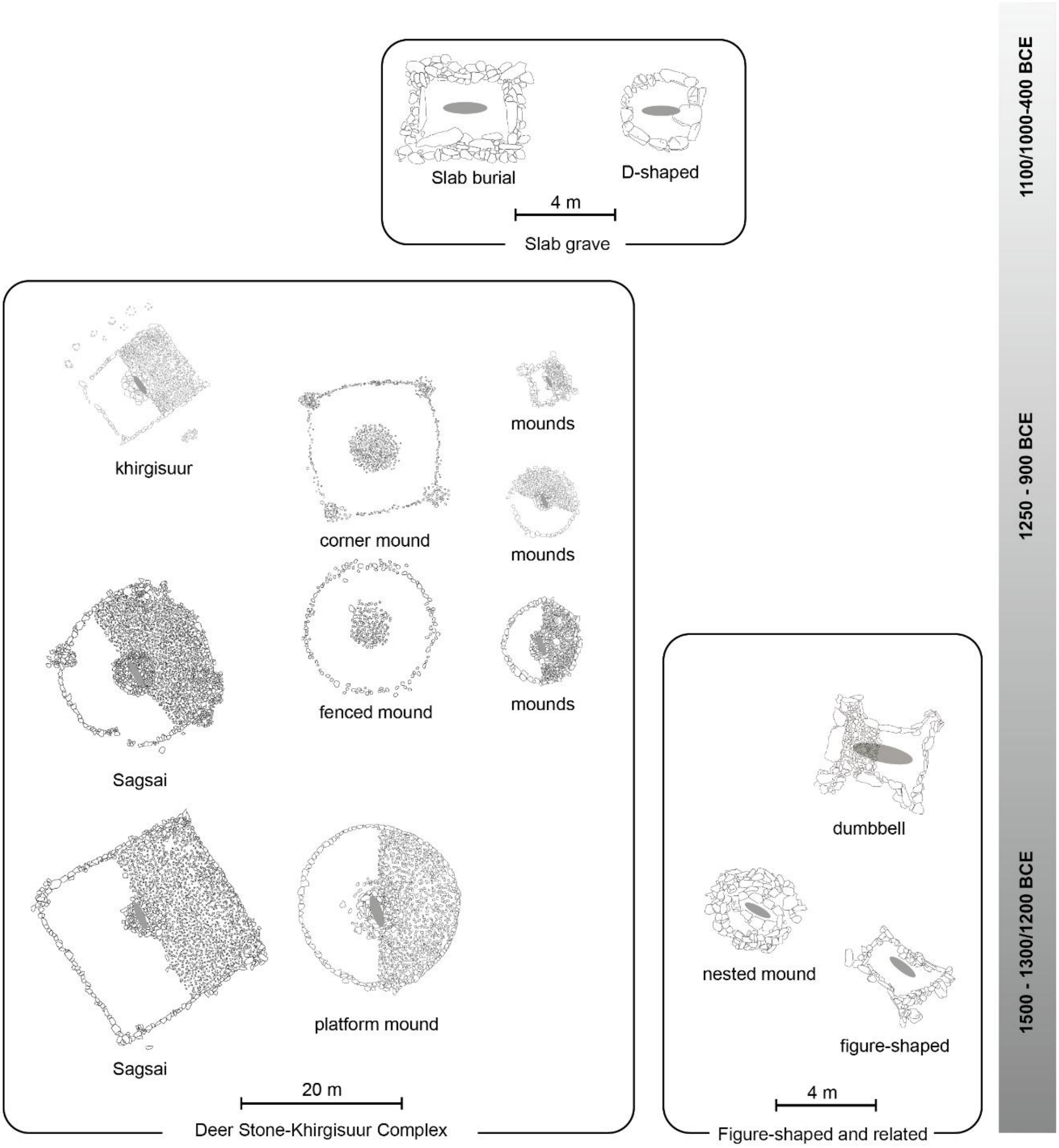

Supplementary Figure 2. Grave forms at Maikhan Tolgoi, central Mongolia. The grave forms at Maikhan Tolgoi in central Mongolia are categorized by cultural macrogroup, with time shown on the right. During the Late Bronze Age, two distinct macrogroups coexisted: Deer Stone-Khirgisuur Complex (DSKC) and figure-shaped burials (and related forms). In the Early Iron Age, Slab Graves replaced these earlier traditions.

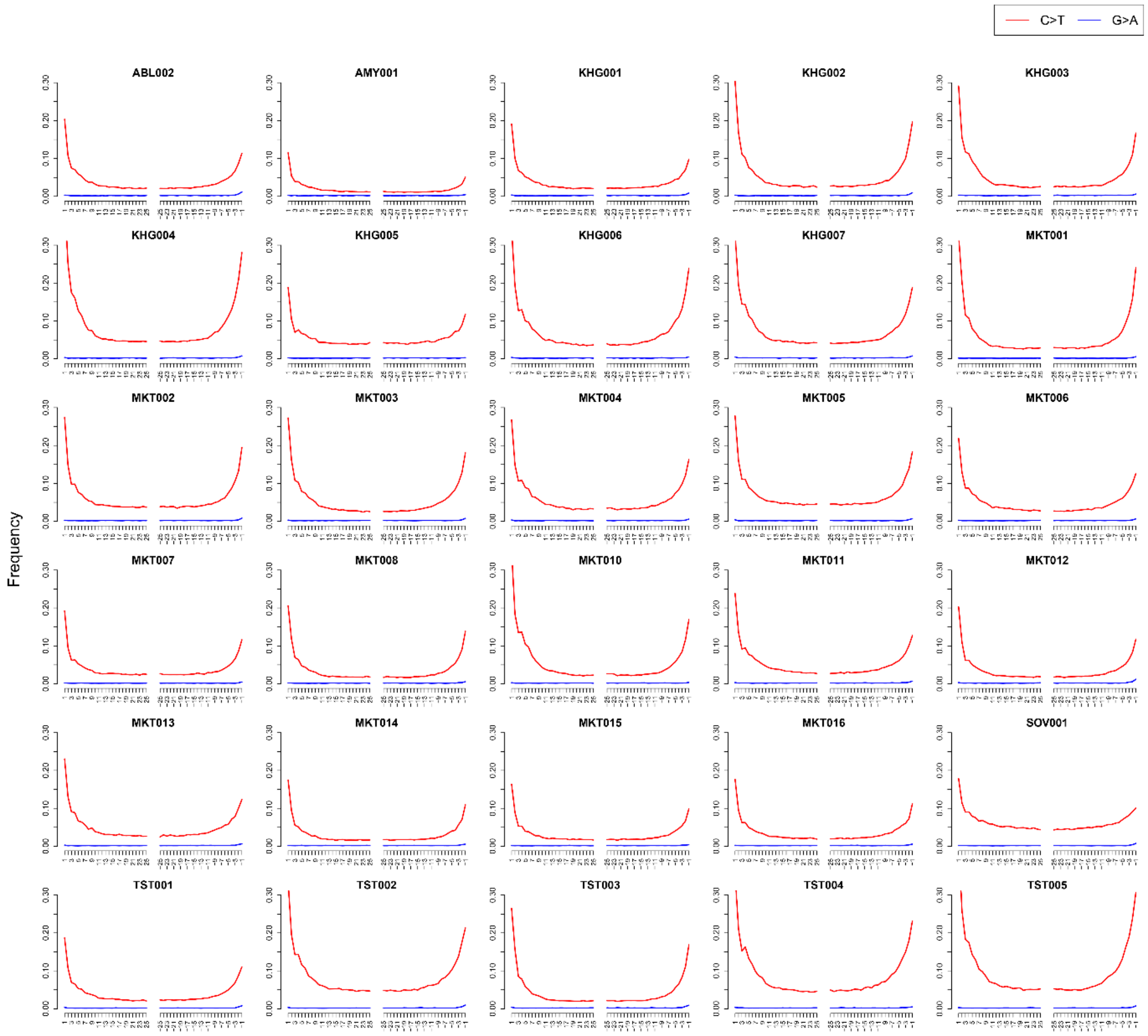

Supplementary Figure 3. The misincorporation patterns of newly sequenced individuals. The misincorporation patterns of 30 newly sequenced individuals from central Mongolia are shown in this figure. The misincorporation patterns were estimated using mapDamage and summarized in two panels for each individual: the left and right panel shows the damage pattern at 5’ and 3’ ends of the reads mapped to hs37d5, respectively. The x-axis represents the base position of the reads. The y-axis represents the frequency of misincorporations. Red lines indicate C>T misincorporations and blue lines indicate the G>A misincorporations. High C>T misincorporations are observed at both ends of the reads, while G>A misincorporations are lower, consistent with the library preparation strategy used for these individuals. Source data are available at https://zenodo.org/records/16743201 [19].

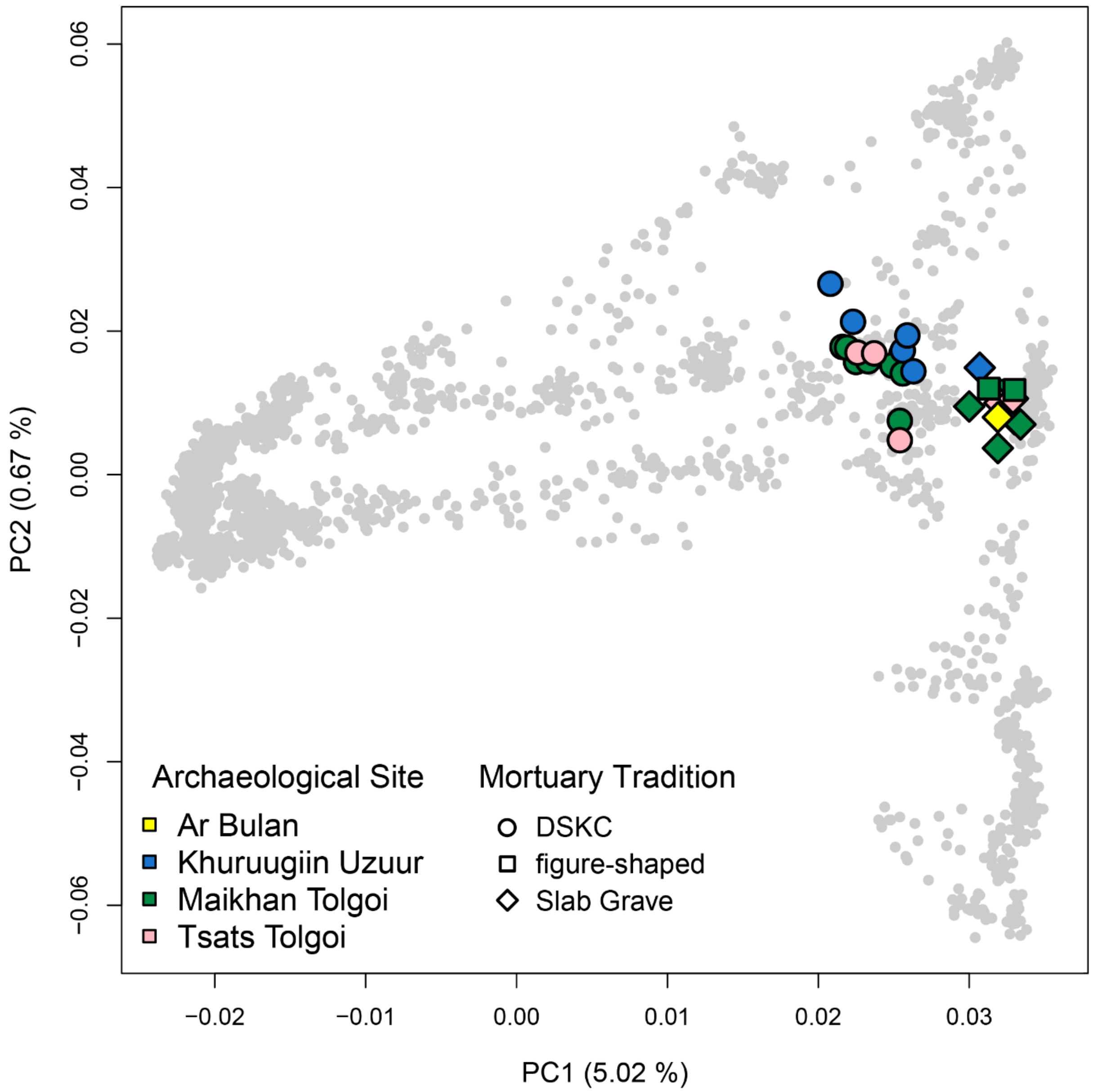

Supplementary Figure 4. The genetic profiles of newly sequenced individuals from LBA/EIA periods in central Mongolia. We conducted Principal Component Analysis (PCA) of the 27 newly analyzed individuals from four archaeological sites in central Mongolia. PCs were calculated using modern Eurasians and ancient individuals were projected on the calculated PCs. Modern Eurasian individuals are represented by gray dots, while the newly analyzed ancient individuals are shown as colored and shaped symbols. The colors and shapes of the symbols represent the archaeological site and the mortuary tradition of each individual, respectively. Notably, Maikhan Tolgoi (MKT) is the only site where individuals from both the DSKC and figure-shaped burial traditions were uncovered. Source data are available at https://zenodo.org/records/16743201 [19].

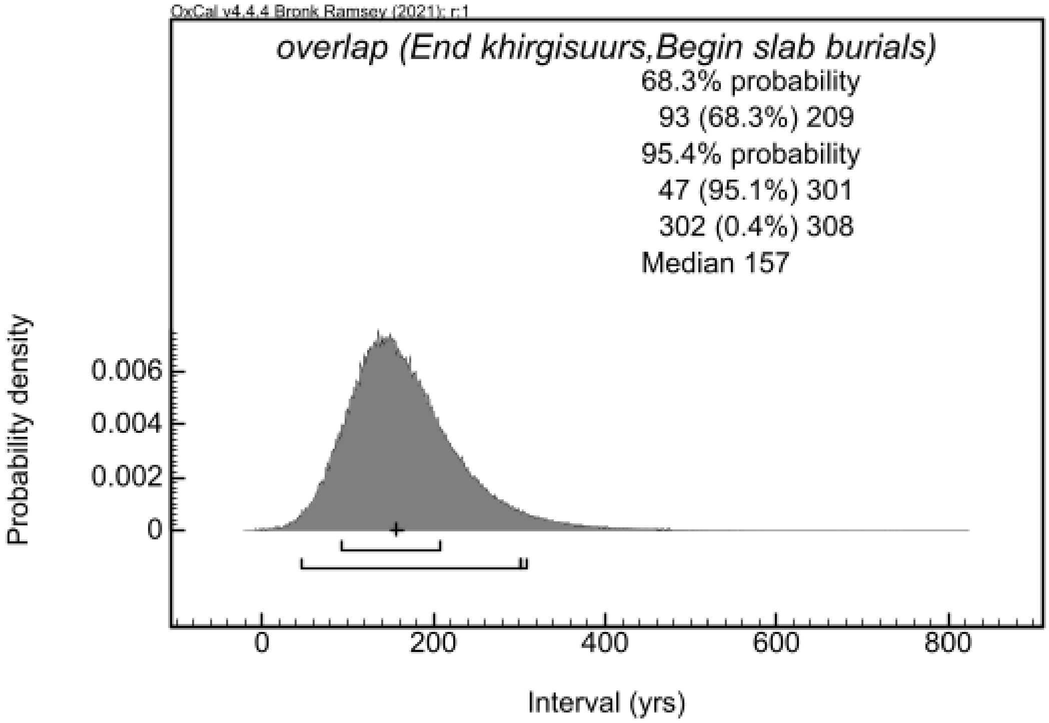

Supplementary Figure 5. Modeled Temporal Overlap Between the End of Khirgisuur and the Beginning of Slab Graves. Bayesian modeling of 24 graves in the Orkhon Valley estimates an overlap of approximately 150 years between Khirgisuur and Slab Grave traditions, with a median overlap of 157 years (95% confidence interval). Source data are available at https://zenodo.org/records/16743201 [19].

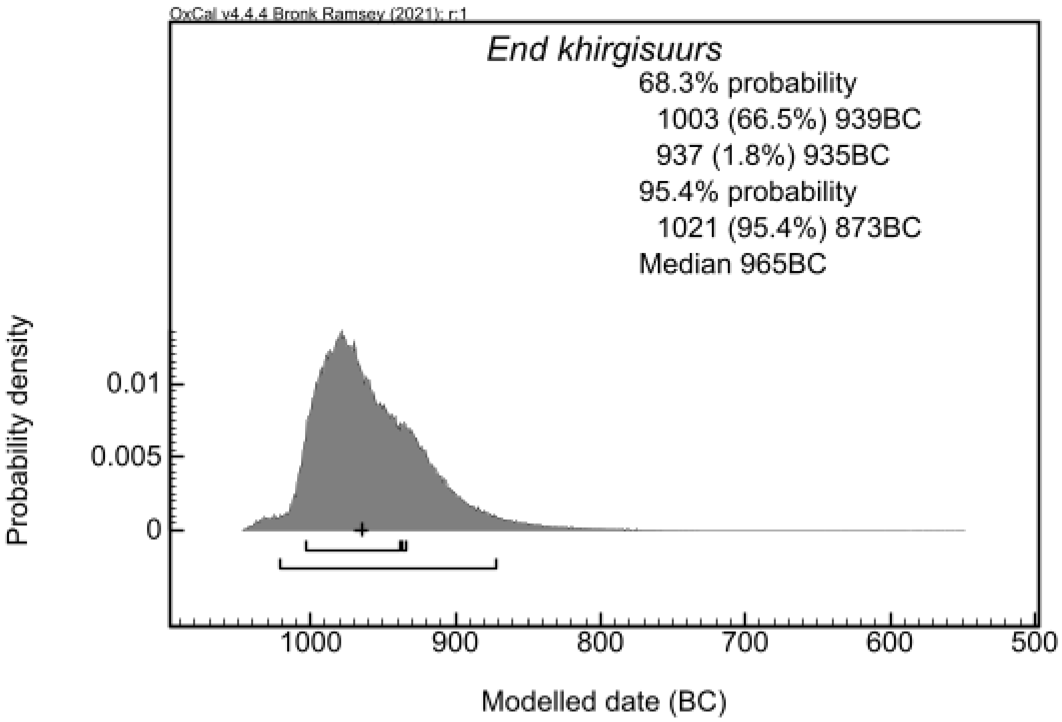

Supplementary Figure 6. Modeled date for the end of the Khirgisuurs in the Orkhon Valley. Bayesian modeling based on 13 Khirgissurs in the Orkhon Valley suggests that Khirgisuur construction ended between 1021 and 873 BCE (95% confidence interval). Source data are available at https://zenodo.org/records/16743201 [19].

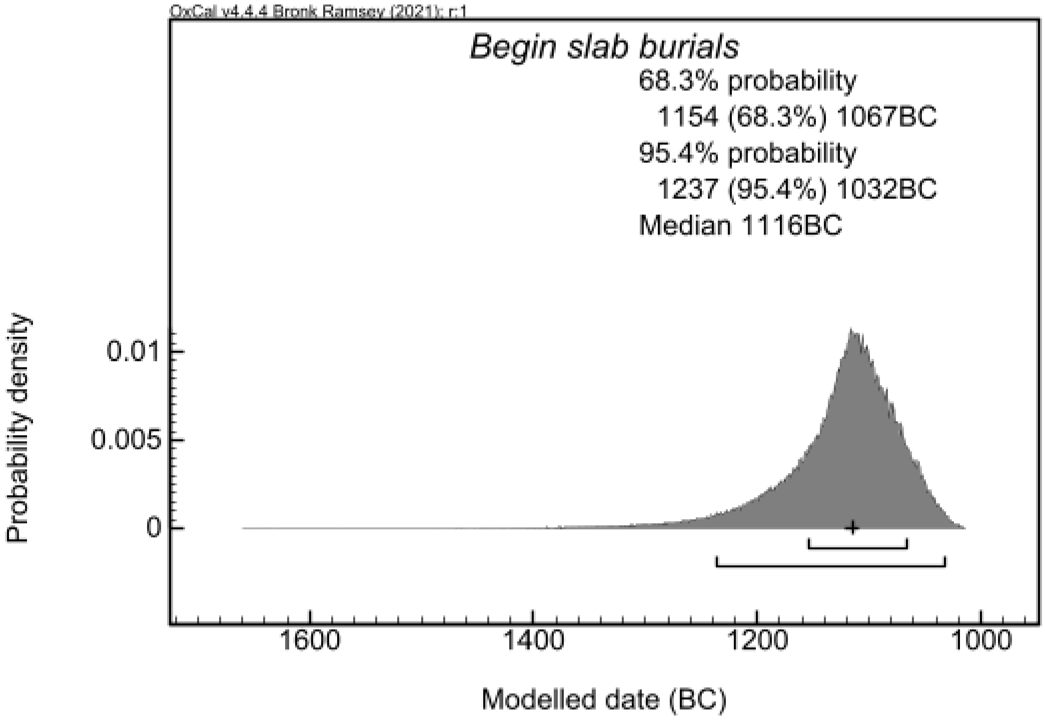

Supplementary Figure 7. Modeled date for the begin of the Slab graves in the Orkhon Valley. Bayesian modeling based on 11 Slab graves in the Orkhon Valley suggests that Slab Grave construction began between 1237 and 1032 BCE (95% confidence interval). Source data are available at https://zenodo.org/records/16743201 [19].

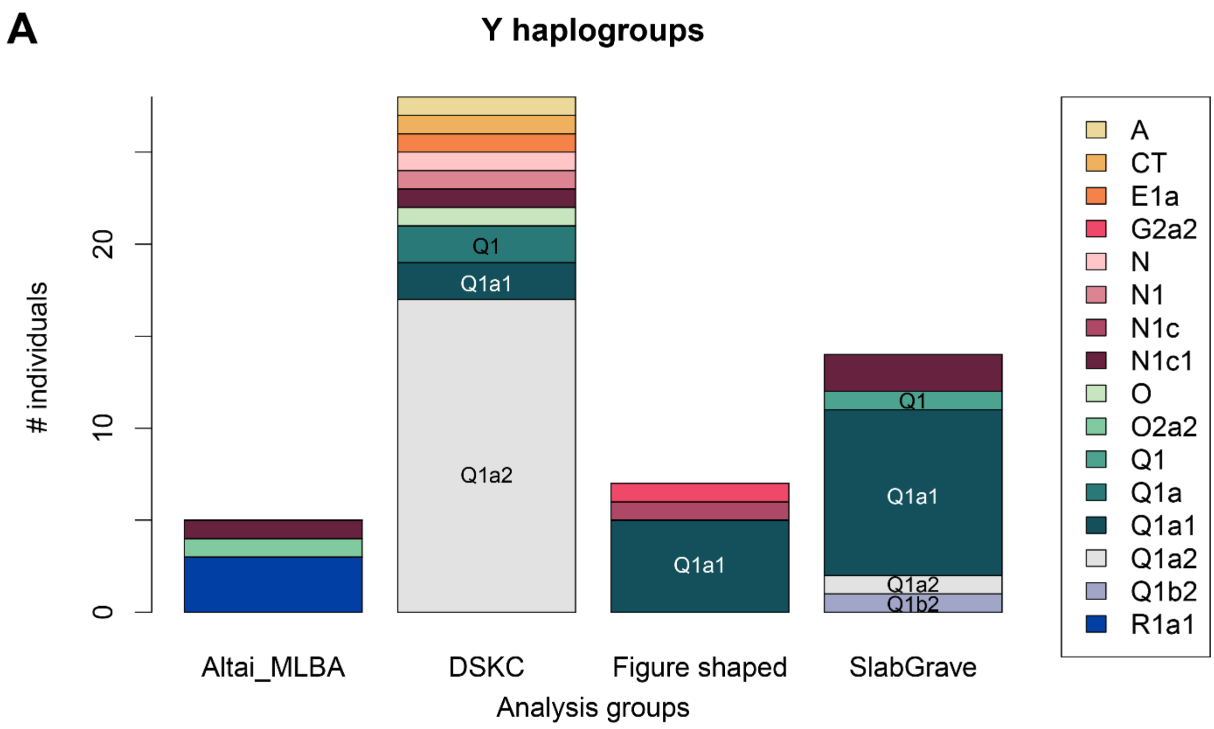

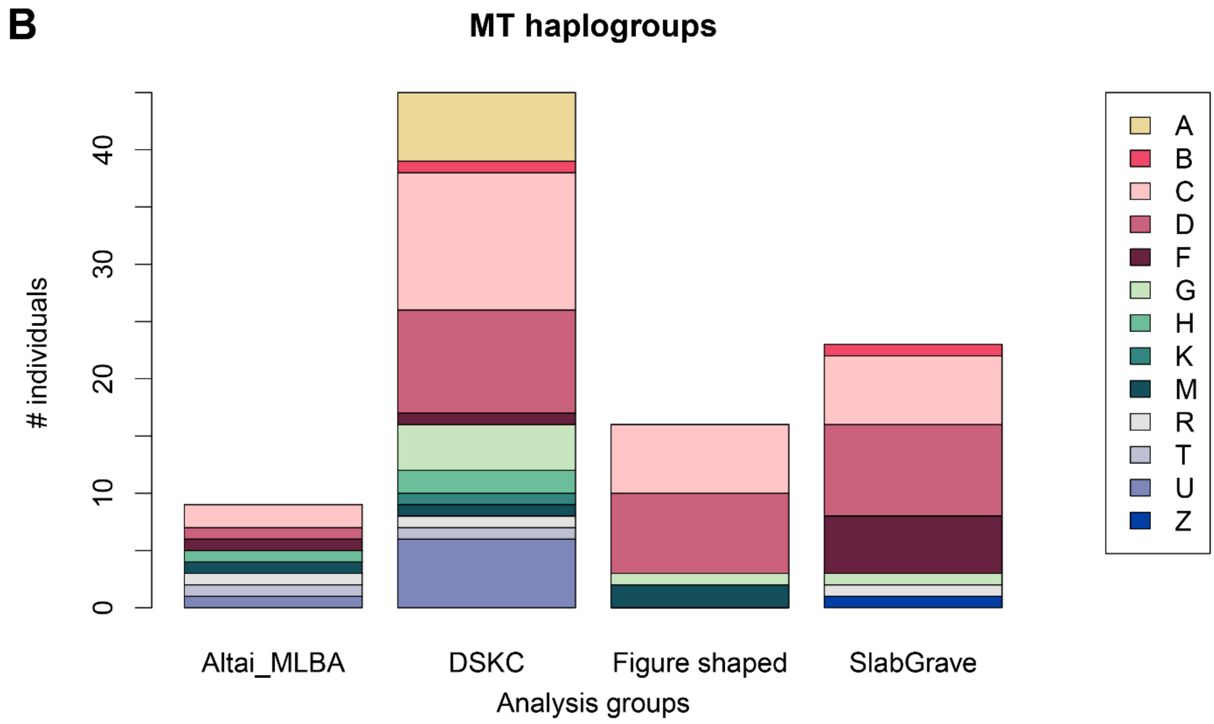

Supplementary Figure 8. Uniparental haplogroup distributions among LBA and EIA pastoralists from Mongolia. Stacked bar plots display the distribution of (A) Y chromosome and (B) mitochondrial (MT) haplogroups across four analysis groups. The number of individuals per group is shown on the y-axis. Colors correspond to distinct haplogroups, as indicated in the legend. Only individuals with assigned haplogroups are included. (A) Y haplogroup composition reveals a clear distinction between groups: Q1a2 is predominant in the DSKC group, while Q1a1 dominates among figure-shaped and SlabGrave individuals. (B) MT composition does not show similarly clear group-specific patterns. Source data are available at https://zenodo.org/records/16743201 [19].

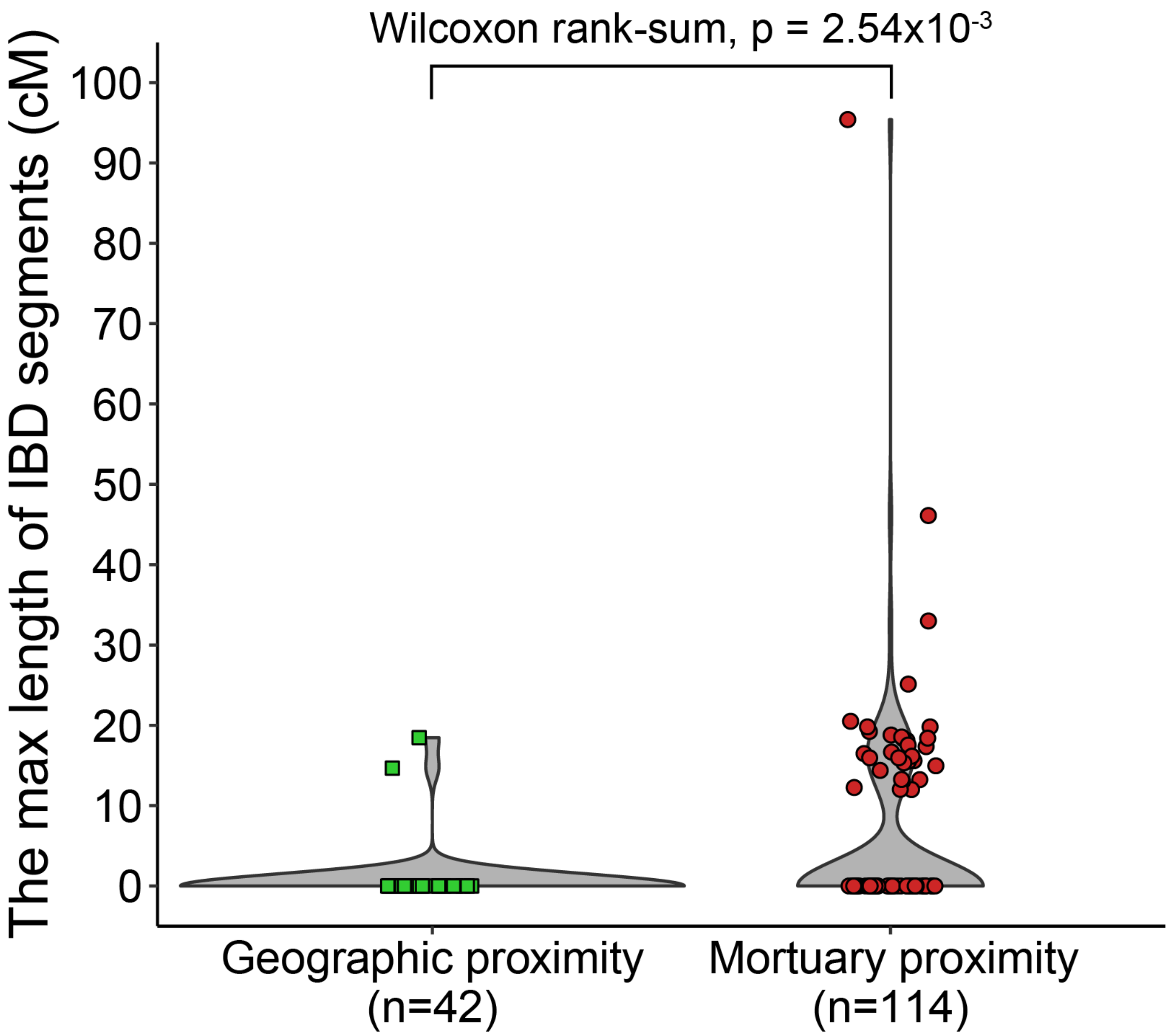

Supplementary Figure 9. Extended Identity by Descent (IBD) sharing between individuals with the same mortuary traditions. This violin plot compares the maximum length of IBD blocks between two groups, geographical proximity and mortuary proximity group. The geographic proximity group consists of pairs of individuals from different mortuary traditions (one DSKC and one figure-shaped), excavated within a 200 km radius. The mortuary proximity group consists of pairs from the same mortuary tradition, but excavated from locations more than 200 km apart. The maximum length of each pair is shown by the colored symbol. The two-sided p-value for the Wilcoxon rank-sum test is shown at the top of the plot. Despite the greater geographic distance, individuals sharing the same mortuary tradition shared more IBD blocks than those buried nearby but from different traditions. Source data are available in Supplementary Data 9.

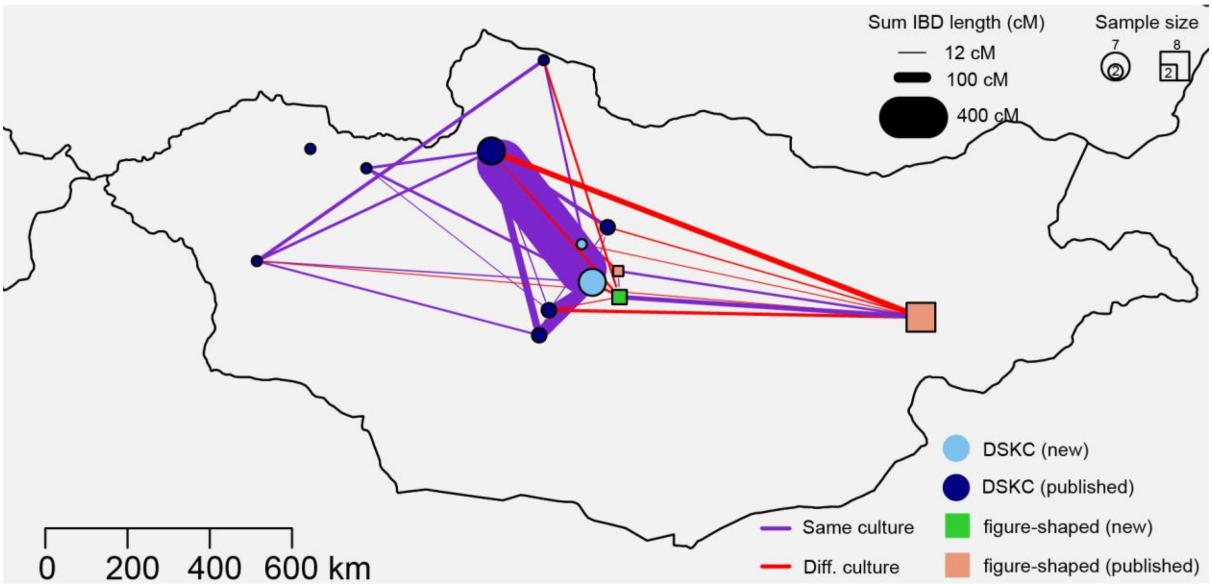

Supplementary Figure 10. IBD blocks among DSKC and figure-shaped individuals on the map. We plotted IBD sharing among individuals associated with DSKC and figure-shaped on the map, by grouping them based on their archaeological sites. Each archaeological site is marked with a colored symbol, where the color and shape represent the mortuary tradition of the site. For sites with more than one mortuary tradition, two symbols are used. The size of each symbol reflects the number of individuals excavated from that site. To improve clarity, the longitude and latitude of overlapping sites have been slightly adjusted, with the modified coordinates provided in Supplementary Data 10. Colored lines represent IBD connections between sites, with purple indicating pairs that share the same mortuary tradition and red indicating pairs with different mortuary traditions. The width of the lines connecting two sites indicate the total length of IBD blocks shared between them. Only IBD blocks that are at least 12 cM in length are displayed on the map. Source data are available in Supplementary Data 7 and 10.

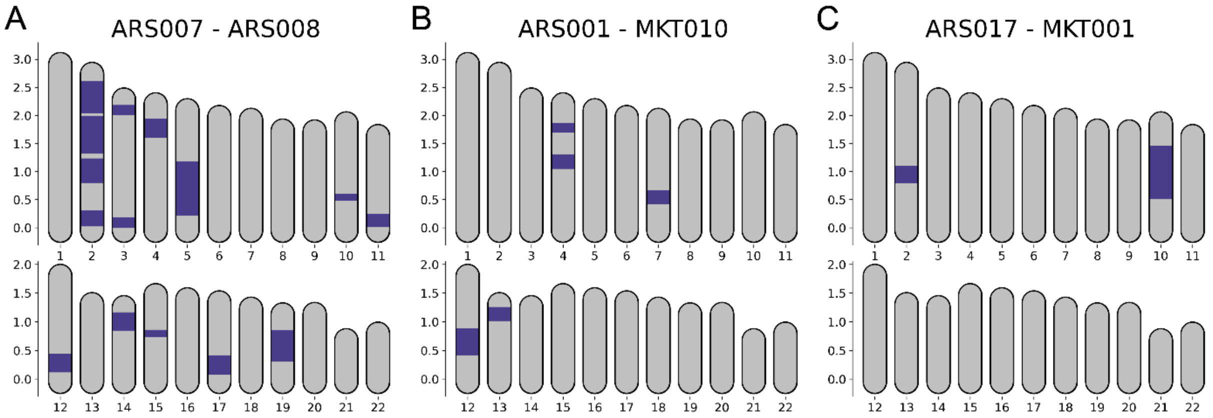

Supplementary Figure 11. Inferred IBD shared between three pairs of individuals sharing more than 100 cM of IBD. Among the 1,711 pairs of individuals analyzed in this study, three pairs are newly identified as genetic relatives, sharing over 100 cM of IBD segments. All shared IBD blocks that are at least 12 cM long are visualized in a karyotype, with the IBD blocks represented by dark blue segments. (A) IBD blocks shared between ARS007 and ARS008. They are estimated to be the 4th degree relatives, based on the ancIBD and KIN analysis. (B) IBD blocks shared between ARS001 and MKT010. They are estimated to be the 5th degree relatives, based on the ancIBD and KIN analysis. (C) IBD blocks shared between ARS017 and MKT001. They are estimated to be the 5th degree relatives, based on the ancIBD and KIN analysis. Source data are available at https://zenodo.org/records/16743201 [19].

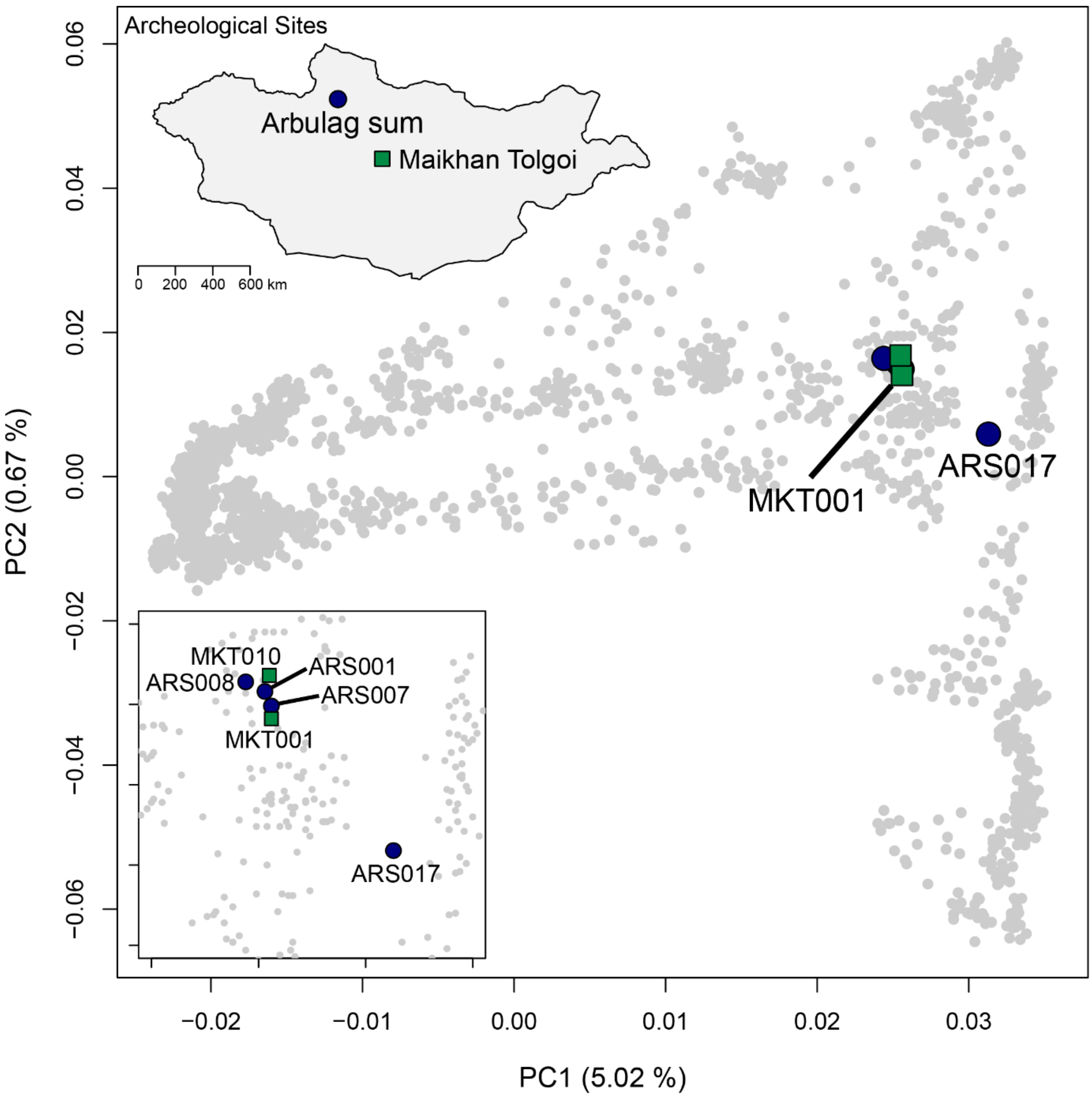

Supplementary Figure 12. Genetic relatives among LBA/EIA individuals in central Mongolia. Among the total of 1,711 pairs of LBA/EIA individuals analyzed in this study, three newly identified pairs are confirmed as genetic relatives based on ancIBD and KIN analysis: ARS001-MKT010, ARS007-ARS008, and ARS017-MKT001. Each pair shares more than 100 cM of IBD blocks and is classified as 4th to 5th degree relatives by KIN analysis. All pairs are associated with the DSKC culture and were excavated from two archaeological sites: Arbulag sum (ARS) and Maikhan Tolgoi (MKT). The geographical locations of the two sites are shown in the inset map of Mongolia. ARS and MKT is located approximately 360 km apart. The genetic profiles of the six individuals are visualized through Principal Component Analysis (PCA). The PCs were calculated using modern Eurasian populations, with the ancient individuals projected on the calculated PCs. Modern Eurasians are represented by gray dots, while the six ancient individuals are indicated by colored symbols, corresponding to their archaeological sites. The inset figure provides a zoomed-in view of the PCA plot, with x-axis ticks marking PC1 from 0.020 to 0.035 and y-axis ticks marking PC2 from 0 to 0.02. Notably, ARS017, a 5th-degree relative of MKT001, exhibits a genetic profile more similar to Ancient Northeast Asians (ANA), which is uncommon among DSKC individuals. Source data are available at https://zenodo.org/records/16743201 [19].

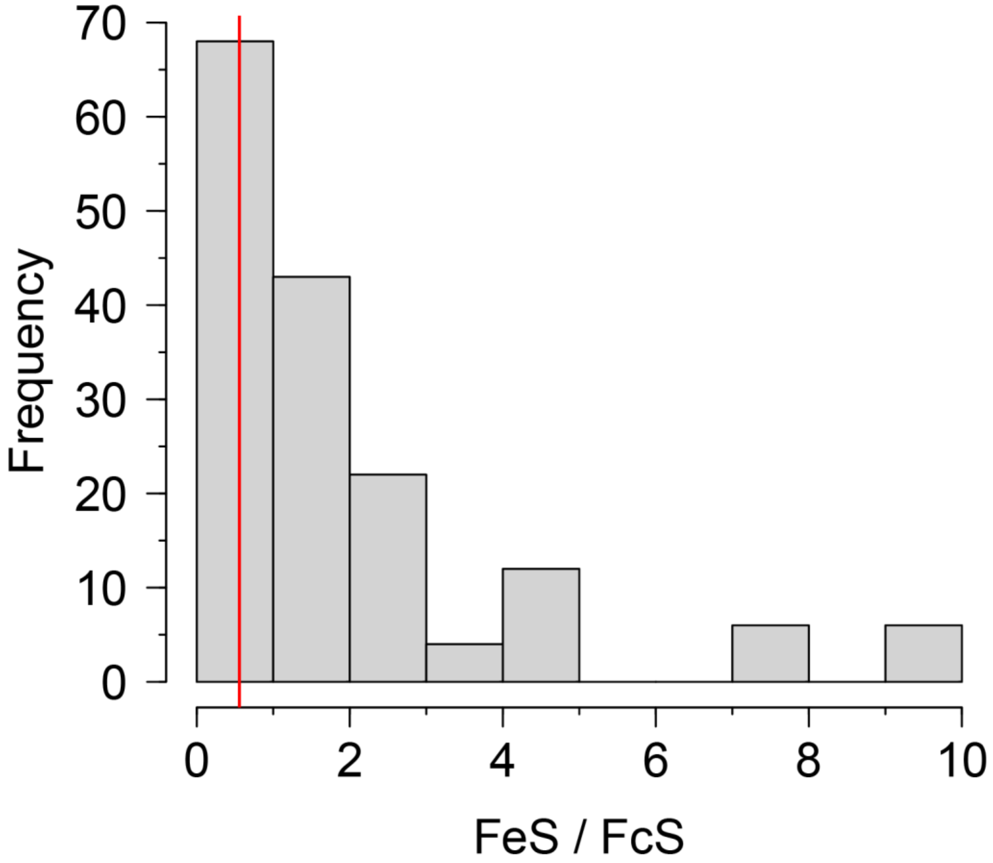

Supplementary Figure 13. The distribution of the IBD enrichments between figure-shaped individuals from eastern Mongolia and Slab Grave individuals, generated by permutations. We tested the archaeological hypothesis that the Slab Grave culture originated from figure-shaped burials in eastern Mongolia by examining the enrichment of IBD shared between these groups. Specifically, we calculated the average IBD shared between figure-shaped individuals from eastern Mongolia (Fe) and Slab Grave individuals (S) (FeS), and compared it to the average IBD shared between figure-shaped individuals from central Mongolia (Fc) and S (FcS). A significantly higher FeS/FcS ratio would suggest a greater genetic contribution from Fe compared to Fc, supporting the hypothesis that the Slab Grave culture originated from figure-shaped burial groups in eastern Mongolia. The ratio was calculated for all possible permutations (n=165) and the distribution of these ratios is shown in the figure. In the single case where FcS = 0, we substituted 0.01 for FcS to prevent division by zero, and excluded this case from the plot for clarity (In that case, FeS / FcS = 288.8). The red vertical line indicates the observed value. Source data are available in Supplementary Data 13.

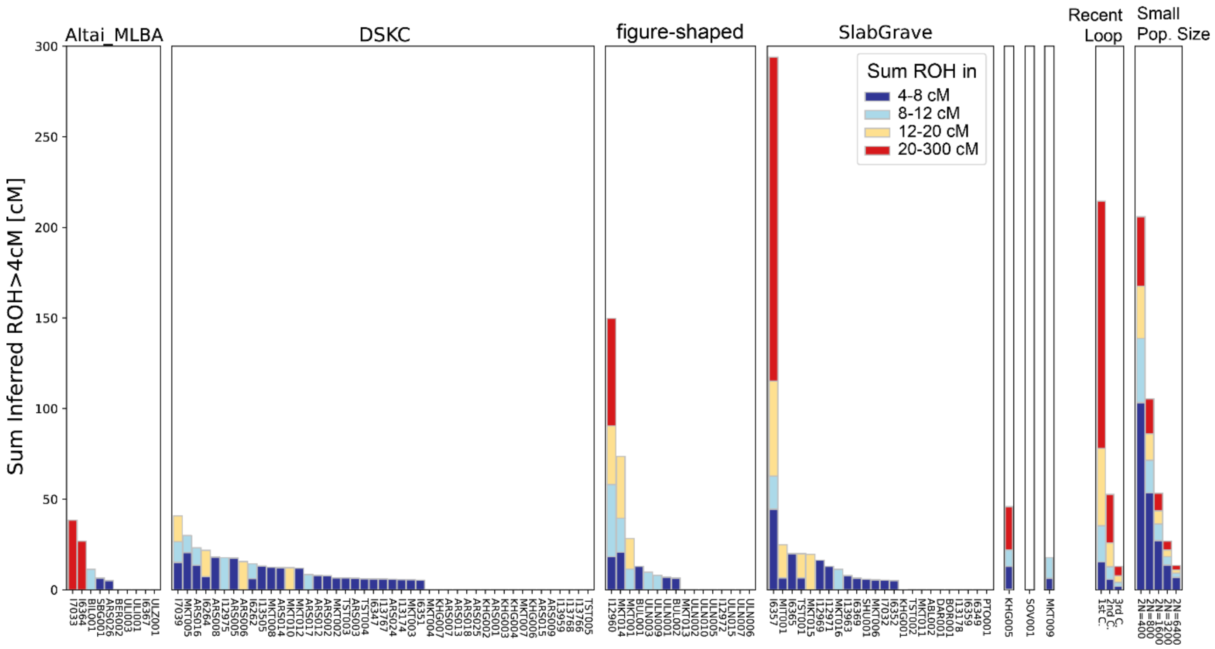

Supplementary Figure 14. HapROH results for LBA and EIA individuals from Mongolia. We analyzed Runs of Homozygosity (ROH) in LBA and EIA individuals from Mongolia, along with three newly generated individuals from subsequent periods, using hapROH. The results are presented according to the analysis groups used in this study. ROH blocks are categorized by length. The ROH blocks longer than 20 cM, shown in red, are usually considered as the signature of the close-kin marriage. Shorter ROH blocks suggest a small population size. The right two panels show expected ROH patterns for close-kin marriage (Recent Loops) and small population sizes (Small Pop. Size). They illustrate expected ROH blocks resulted from the marriage between the first cousins (1st C.), second cousins (2nd C.), and third cousins (3rd C.) and the small population size with 400 (2N=400), 800 (2N=800), 1,600 (2N=1600), 3,200 (2N=3200) and 6,400 (2N=6400) individuals. Source data are available at https://zenodo.org/records/16743201 [19].

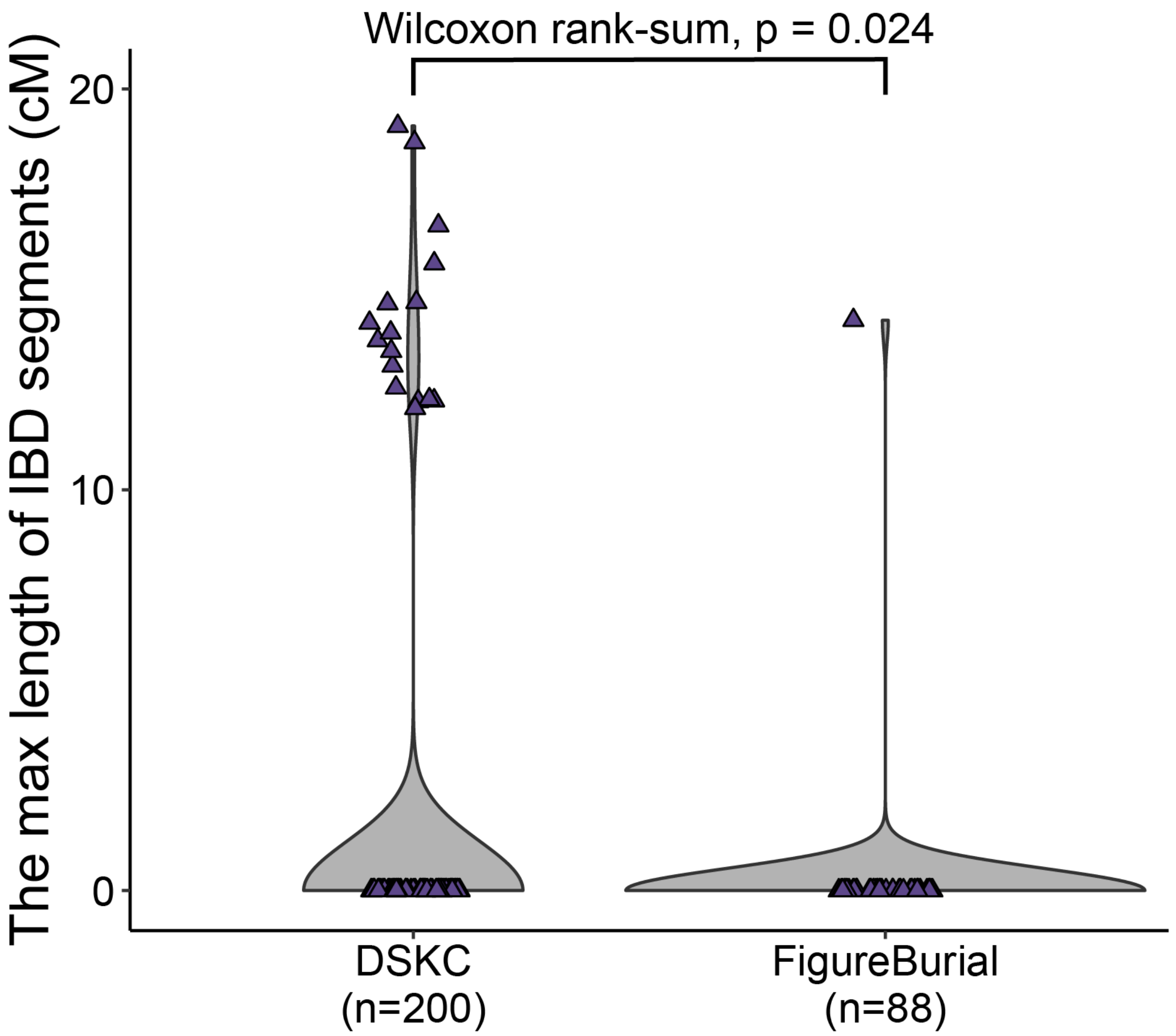

Supplementary Figure 15. Extended Identity by Descent (IBD) sharing of Altai_MLBA individuals with Mongolia_LBA_DSKC than with figure-shaped individuals. This violin plot compares the maximum length of IBD blocks of Altai_MLBA individuals (n=8) shared with DSKC (n=25) and figure-shaped individuals (n=11). The maximum length of each pair is shown as the colored symbols. The two-sided p-value for the Wilcoxon rank-sum test is shown at the top of the plot. Consistent with the qpAdm modeling of Altai_MLBA individuals, which are explained as the mixture of Mongolia_LBA_DSKC and the western steppe ancestry, and the shared mortuary tradition of DSKC culture, Altai_MLBA individuals shared more IBD blocks with Mongolia_LBA_DSKC than with figure-shaped individuals. Source data are available in Supplementary Data 7.

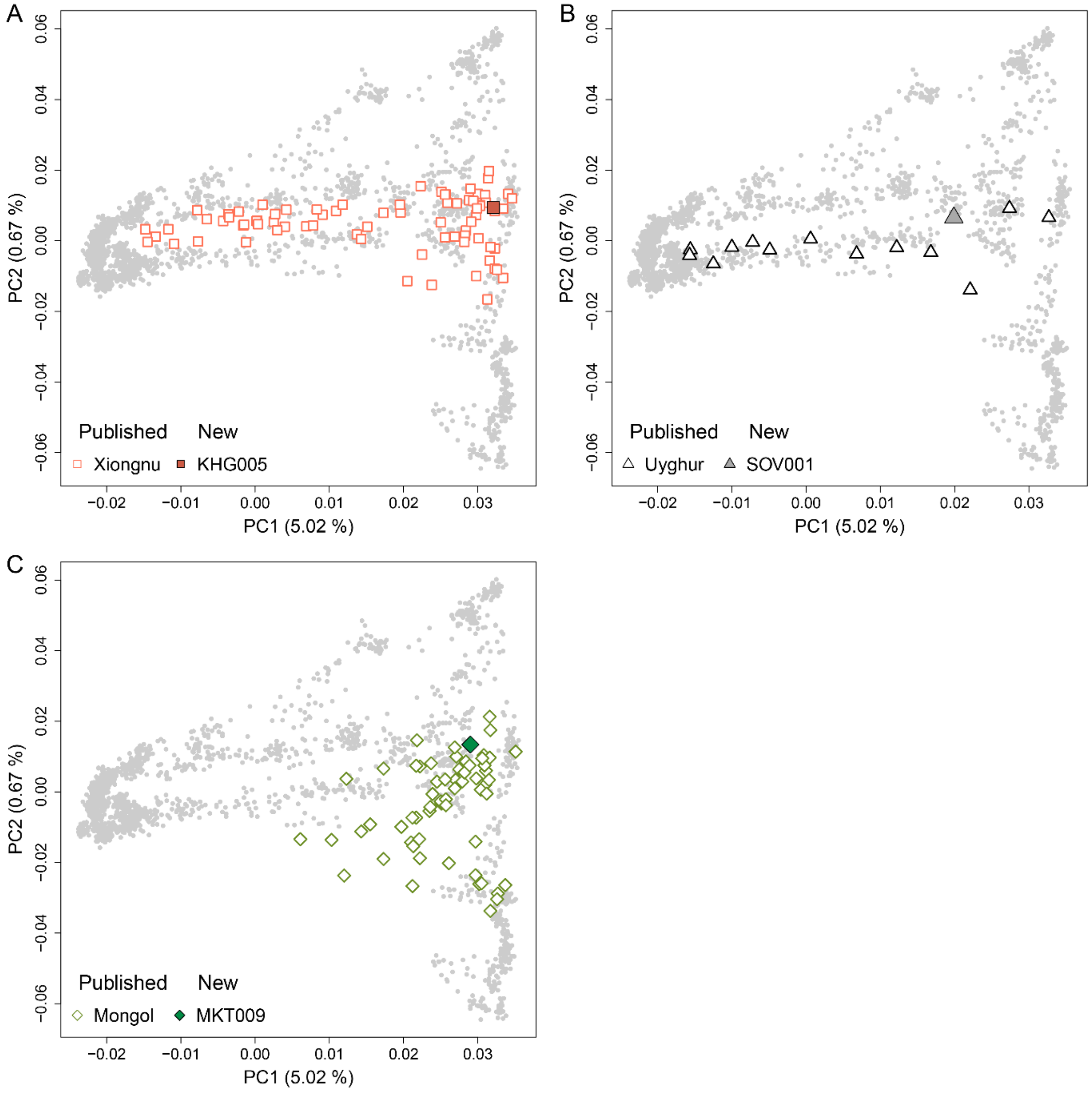

Supplementary Figure 16. The genetic profiles of newly sequenced individuals from the Xiongnu, Uyghur, and Mongol Empire periods in central Mongolia. We conducted PCA of the three newly analyzed individuals associated with the Xiongnu, Uyghur and Mongol empire. PCs were calculated using modern Eurasians and ancient individuals were projected on the calculated PCs. Modern Eurasian individuals are represented by gray dots, while the previously published individuals and newly analyzed ancient individuals are shown in empty and colored symbols, respectively. The color and shape of each symbol denote the period of the individuals: red squares for Xiongnu individuals, gray triangles for Uyghur individuals and green diamonds for Mongol individuals. (A) Xiongnu individuals are plotted on the PCA of the modern Eurasians. (B) Uyghur individuals are plotted on the PCA of the modern Eurasians. (C) Mongol individuals are plotted on the PCA of the modern Eurasians. Source data are available at https://zenodo.org/records/16743201 [19].

Supplementary References

1 Bemmann, J., Batsükh, D., Gantulga, J.-O., Yeruul-Erdene, C. & Brosseder, U. *Searching for patterns through the ages in ritual landscapes of Bronze Age Mongolia*. in *Man sieht nur, was man weiß, man weiß nur, was man sieht. Globalhistorische Perspektiven auf interkulturelle Phänomene der Mobilität. Festschrift für Hermann Parzinger zum 65. Geburtstag* (eds J. Schneeweiss, M. Nawroth, H. Piezonka, & H. Schwarzberg) 605-628 (2024).

2 Chunag, A. *Türüülge n' kharuulsan orshuulgat bulsh*. in *Arkheologiin sudalgaa: Mongolchuudyn garal, ugsaa-soelyn khögzhil.* (eds A. Ochir & Ts. Turbat) 22-50 (Mönkhiin üseg KhKhK, 2020).

3 Gantulga, J., Amartuvshin, C. & Turbat, T. Conceptualization of a new type of funeral-ritual monument of the Mongolian Iron Age. *in press*

4 Kovalev, A. A. & Erdenebaatar, D. *Discovery of new cultures of the Bronze Age in Mongolia according to the data obtained by the International Central Asian Archaeological Expedition*. in *Papers from the First International Conference on "Archaeological Research in Mongolia" held in Ulaanbaatar, August 19th–23rd, 2007.* Vol. Bonn Contributions to Asian Archaeology 4 (ed H. Parzinger J. Bemmann, E. Pohl, D. Tseveendorzh) 149-170 (Vfgarch, 2009).

5 Frohlich, B., Amgalantugs, T., Littleton, J., Hunt, D. & Goler, K. *Bronze Age Burial Mounds in the Khovsgol aimag, Mongolia.* in *Current Archaeological Research in Mongolia. Papers from the First International Conference on "Archaeological Research in Mongolia" held in Ulaanbaatar, August 19th–23rd, 2007.* Vol. Bonn Contributions to Asian Archaeology 4 (ed H. Parzinger J. Bemmann, E. Pohl, D. Tseveendorzh) 99-116 (Vfgarch, 2009).

6 Frohlich, B. *et al. Theories and hypotheses pertaining to Mongolian Bronze Age khirigsuurs in Hovsgol amaig, Mongolia*. in *American-Mongolian Deer Stone Project: Field Report 2009* (eds W. Fitzhugh & J. Bayarsaikhan) 195-210 (2010).

7 Turbat, T. *et al. Mongol ba bus nutgiin bugan khöshöönii soyol. III.* (Mönkhiin useg, 2021).

8 Houle, J.-L. *Emergent complexity on the Mongolian steppe: mobility, territoriality, and the development of early nomadic polities*, University of Pittsburgh, (2010).

9 Turbat, T. *Khemtsegiin soelyn bulsh*. in *Mongolyn ertnii bulsh orshuulga* (ed G. Eregzen) 36-43 (2016).

10 Gantulga, J. *Sagsai khelberiin bulsh*. in *Mongolyn ertnii bulsh orshuulga* (ed G. Eregzen) 58-62 (2016).

11 Gantulga, J. *Sagsai khelberiin bulsh*. in *Arkheologiin sudalgaa: Mongolchuudyn garal, ugsaa-soelyn khögzhil.* (eds A. Ochir & Ts. Turbat) 9-21 (Mönkhiin üseg KhKhK, 2020).

12 Hollard, C. *et al.* Strong genetic admixture in the Altai at the Middle Bronze Age revealed by uniparental and ancestry informative markers. *Forensic Science International: Genetics* **12**, 199-207 (2014).

13 Turbat, T. Tsagaan Asgyn Pazyrykiin üeiin bulshny sudalgaany ur'dchilsan ür dün. *Studia Archaeologica* **26**, 115-135 (2008).

14 Gantulga, J., Eruul-Erdene, C., J. Magai, K. S., Lkhundev, G. & Bazargur, D. *Khoid Tamiryn khöndii dekh maltlaga sudalgaa (Khoid Tamiryn khöndii dekih arkheologiin sudalgaa-III).* (Soembo printing, 2023).

15 Ganbaatar, G. & Daisuke, N. Arkheologiin dursgal neriin gazhuudal: Dornod Mongolyn khürel ba türüü tömriin üeiin arkheologiin dursgalyn zhisheen deer. *Studia Archaeologica* **42**, 108-114 (2023).

16 Erdenebaatar, D. & Kovalev, A. Tevshiin soel. *Niigmiin shinjlekh ukhaany erdem shinjilgeenii bichig* **3**, 4-38 (2008).

17 Honeychurch, W. *Inner Asia and the spatial politics of empire: archaeology, mobility, and culture contact*. (Springer, 2015).

18 Miyamoto, K., Adachi, T., Amgalantgus, T. & Batbold, N. *Excavations at Emeelt Tolgoi Site: The third Report on Joint Mongolian-Japanese Excavations in Outer Mongolia*. (2018).

19 Lee, J., Brosseder, U. & Moon, H. CWJeongLab/Central_Mongolia: Central_Mongolia_v01. Zenodo https://zenodo.org/records/16743201 (2025).
