# Supplementary material for: Slab Grave expansion disrupted long co-existence of distinct Bronze Age herders in central Mongolia
Source: Nat Commun. 2025 Sep 25;16:8420. doi: 10.1038/s41467-025-63789-1 (PMC12462455; doi:10.1038/s41467-025-63789-1)
Supplement: Supplementary file 2 — Description of Addtional Supplementary Files [file 41467_2025_63789_MOESM2_ESM.pdf]

Supplementary Data 1. Summary of the individuals newly reported in this study. We generated DNA libraries for the total of 32 individuals and 30 of them yielded sufficient amount of human DNA (>0.09% reads mapped on hs37d5) for the genomic analysis. These 30 libraries were enriched for 1,233,013 nuclear SNPs (1240K) and sequenced (1240K capture sequencing). We also generated and sequenced whole-genome shotgun sequencing libraries for 10 individuals with ≥10% human DNA (Shotgun deep sequencing). Archaeological details and sequencing summaries for all 32 individuals are provided below.

Supplementary Data 2. The published populations used for the population genetic analysis. "O" means that the corresponding population is an ancient one ("Ancient"), is included in a data set (1240K/HO) or used for the corresponding analysis.

Supplementary Data 3. List of LBA and EIA Analysis Groups used in this study. A total of 83 individuals were excavated from burials that belong to one of three representative burial traditions in Mongolia (Figure-shaped, DSKC/sagsai, and Slab Grave burials) and exhibit one of two typical genetic profiles (ANA-like and Khovsgol_LBA-like genetic profiles). These individuals were divided into several analysis groups based on their cultural affiliations and genetic profiles. Generally, a clear correlation was observed between archaeological affiliation and genetic profiles, but some outliers exhibited mismatched archaeological and genetic profiles. (A) List of analysis groups for 58 previously published individuals affiliated with Figure-shaped, DSKC/sagsai, and Slab Grave burial cultures, showing ANA-like or Khovsgol_LBA-like genetic profiles. These individuals were divided into six groups, with the name of the group and the number of individuals in each group listed in the supplementary data. (B) List of previously published individuals affiliated with Figure shaped, DSKC/sagsai, and Slab Grave burial cultures (n=67). Of these, 58 individuals were divided into six analysis groups as shown in the supplementary data, while the remaining 9 individuals belong to the Altai_MLBA group. (C) List of analysis groups for 25 newly reported individuals affiliated with Figure shaped, DSKC/sagsai, and Slab Grave burial cultures, showing ANA-like or Khovsgol_LBA-like genetic profiles. These individuals were divided into six groups, with the name of the group and the number of individuals in each group listed in the supplementary data.

Supplementary Data 4. QpWave and qpAdm modeling of 27 newly sequenced individuals from central Mongolia. We conducted the qpWave and qpAdm analysis of 27 newly sequenced individuals from central Mongolia. Models with qpWave/qpAdm p-values >0.05 are highlighted in grey and models presented in the main text are highlighted in bold. We used a base set of ten outgroups ("Base"): Mbuti, Onge, Natufian, Iran_N, Villabruna, Ami, Mixe, Anatolia_N as a default. (A) Summary of the group-based qpWave analysis of 25 individuals from central Mongolia. We performed group-based qpWave analysis of CentralMongolia_LBA_DSKC (n=15), CentralMongolia_LBA_FigureBurial (n=3) and CentralMongolia_EIA_SlabGrave (n=8). We found that three populations are genetically indistinguishable with the previously published Khovsgol_LBA, Ulaanzuukh1 and SlabGrave1, respectively, consistent with their archeological affiliations. (B) Summary of the individual-based qpAdm analysis of 25 individuals from central Mongolia. We performed individual-based qpWave analysis of 27 newly sequenced individuals using the Base outgroups. (C) Summary of the group-based qpWave/qpAdm analysis of CentralMongolia_LBA_DSKC_outlier. We performed the group-based qpWave/qpAdm analysis of CentralMongolia_LBA_DSKC_outlier (n=2) using the Base outgroups. (D) Summary of the individual-based qpWave/qpAdm analysis of CentralMongolia_LBA_DSKC_outlier. We performed the individual-based qpWave/qpAdm analysis of CentralMongolia_LBA_DSKC_outlier (n=2) using the Base outgroups.

Supplementary Data 5. List of Middle/Late Bronze Age and Early Iron Age individuals with the cultural affiliations and Y and mitochondrial (MT) haplogroups. The total of 104 individuals (61 males and females) are sorted by their respective analysis groups. (A) List of Y haplogroups of MLBA and EIA males (n=61). The total of 61 males from Middle/Late Bronze Age and Early Iron Age Mongolia with their analysis groups, cultural affiliations, and Y haplogroups are shown in the supplementary data. Typically, individuals associated with the same cultural affiliation are assigned to the same Y haplogroup. (B) List of MT haplogroups of MLBA and EIA individuals (n=104). The total of 104 individuals from Middle/Late Bronze Age and Early Iron Age Mongolia with their analysis groups, cultural affiliations, and MT haplogroups are shown in the supplementary data. No significant differences in MT haplogroups are observed across cultural affiliations.

Supplementary Data 6. List of individuals used for ancIBD analysis. We used the ancIBD program to detect Identity by Descent (IBD) blocks shared among individuals belonging to Mongolia_LBA_DSKC, which includes CentralMongolia_LBA_DSKC and DSKC with ANA-like genetic profiles, CentralMongolia_LBA_FigureBurial, Ulaanzuukh1, CentralMongolia_EIA_SlabGrave, SlabGrave1, SlabGrave2 and Altai_MLBA. Out of a total of 95 individuals, 59 with sufficient coverage (≥0.25X for shotgun sequencing data and ≥1X for capture data) were included in the ancIBD analysis. The IDs and analysis groups of these individuals are listed below.

Supplementary Data 7. Summary of ancIBD results. We detected Identical By Descent (IBD) blocks shared among a total of 60 individuals belonging to Mongolia_LBA_DSKC (n=25), which includes CentralMongolia_LBA_DSKC (n=8), and DSKC with ANA-like genetic profiles (n=2), CentralMongolia_LBA_FigureBurial (n=2), Ulaanzuukh1 (n=9), CentralMongolia_EIA_SlabGrave (n=4), SlabGrave1 (n=9), SlabGrave2 (n=2), and Altai_MLBA (n=8) using the ancIBD program. The supplementary data provides a list of individual pairs along with the number, maximum length, and total sum of IBD blocks shared, categorized by length bins. To reduce false positives, only IBD blocks longer than 12 centimorgans (cM) were included in the analysis. The pairs are sorted by total shared IBD block length, with three pairs sharing over 100 cM of IBD blocks highlighted in bold, indicating close genetic relationships.

Supplementary Data 8. Pairwise matrix for IBD sharing among 60 individuals from LBA and EIA Mongolia. We detected Identical By Descent (IBD) blocks shared among a total of 60 individuals belonging to Mongolia_LBA_DSKC (n=25), which includes CentralMongolia_LBA_DSKC (n=8), and DSKC with ANA-like genetic profiles (n=2), CentralMongolia_LBA_FigureBurial (n=2), Ulaanzuukh1 (n=9), CentralMongolia_EIA_SlabGrave (n=4), SlabGrave1 (n=9), SlabGrave2 (n=2), and Altai_MLBA (n=8) using the ancIBD program. Here, we summarized the sum of IBD blocks (centimorgans, cM) shared between each pair of individuals and presented the results in matrix format. Only IBD blocks longer than 12 cM were considered to minimize false positives. The genetic analysis group and the cultural background of each individual is also shown in the matrix. The IBD blocks shared between individuals with the same cultural affiliation are shown in grey.

Supplementary Data 9. Patterns of IBD block sharing between cultural and geographic proximity groups. To investigate the genetic interactions between the two LBA cultures, DSKC and figure-shaped cultures, during their coexistence in central Mongolia, we analyzed patterns of IBD sharing between

two groups: (1) pairs of individuals where one is from central Mongolia and the other is from different geographical locations but with the same cultural affiliation (the "cultural proximity" group), and (2) pairs of individuals from the same geographical location within central Mongolia but with different cultural affiliations (the "geographic proximity" group). We considered locations within 200 km of each other as the same geographical area. To minimize false positives, only IBD blocks longer than 12 centimorgans (cM) are used for the downstream analysis. The pairs of individuals with the maximum length of IBD blocks, the sum of IBD blocks and the geographical distance between two sites are listed below. The final column shows whether the pair belongs to the geographic or cultural proximity group (n=42 and n=114, respectively). We found that the cultural proximity group shared more IBD blocks than the geographic proximity group, suggesting limited genetic interaction between the two LBA cultures, even at their geographical intersection in central Mongolia.

Supplementary Data 10. List of DSKC and FigureBurial Individuals with Geographic Locations Used in Supplementary Figure 6. We analyzed IBD sharing patterns among individuals associated with DSKC (n=25) and FigureBurial (n=11) by grouping them based on their cultural affiliation and geographic location, and plotted the IBD connections between pairs of sites on the map (Supplementary Figure 6). For sites with overlapping locations, we slightly adjusted their latitude and longitude. The original and modified coordinates used in Supplementary Figure 6 are provided below.

Supplementary Data 11. Summary of KIN results of Late Bronze Age and Early Iron Age individuals associated with DSKC, figure-shaped, and SlabGrave culture. We analyazed the genetic relationships among 96 individuals from Late Bronze Age and Early Iron Age Mongolia asoociated with DSKC, figure-shaped, and SlabGrave culture, using KIN. We reported log likelihood of 10 relationships: unrelated, 5th, 4th, and 3rd degree relatives, grandparent-grandchild, half-sibling, avuncular, sibling, parent-child, and identical. The most likely relationships, indicated by the highest log-likelihood, are highlighted in red. Relationships within 1 or 2 log-likelihood units of the maximum are shown in orange and green, respectively. (A) Summary of KIN analysis for pairs sharing more than 100 cM of IBD blocks. Among 4,560 pairs analyzed, we only highlighted three pairs of individuals sharing more than 100 cM of IBD blocks in the main text, as these levels of sharing provide additional support for the KIN results. All three pairs are associated with the DSKC culture. One pair of related individuals comes from the same site, while the other two pairs consist of individuals from different sites. (B) Summary of KIN analysis. List of the total of 4,560 pairs analyzed. A list of all 4,560 pairs analyzed, with the three pairs highlighted in the main text shown in bold.

Supplementary Data 12. QpWave and qpAdm modeling of ARS017. We conducted the qpWave and qpAdm analysis of ARS017, an individual affiliated with the DSKC culture but exhibiting an ANA-like genetic profile. Models with qpWave/qpAdm p-values > 0.05 are highlighted in grey. Analyses used a default set of ten outgroups ("Base"): Mbuti, Onge, Natufian, Iran_N, Villabruna, Ami, Mixe, and Anatolia_N. (A) Summary of the qpWave analysis of ARS017. Individual-based qpWave analysis using the Base outgroups shows that ARS017 is genetically indistinguishable from previously published Ulaanzuukh1 and SlabGrave1 individuals but clearly distinct from Khovsgol_LBA. (B) Summary of the qpAdm analysis of ARS017. We tested whether ARS017 can be modeled as a mixture of ANA-like ancestry and Khovsgol_LBA using the Base outgroups. Comparison between one-way and two-way models indicates that including Khovsgol_LBA as a second source does not significantly improve model fit. Nested P-values are shown in the final column.

Supplementary Data 13. IBD sharing among figure-shaped and SlabGrave individuals. To investigate the rapid expansion and population replacement by the Slab Grave culture, we analyzed IBD sharing between individuals associated with figure-shaped and Slab Grave burial traditions. We computed three IBD enrichment statistics to evaluate whether the observed IBD patterns are consistent with the hypothesis of a rapid expansion and population replacement by the Slab Grave culture, and tested their statistical significance using permutation-based approaches. (A) IBD Sharing Matrix of figure-shaped and Slab Grave individuals. The matrix shows the sum of IBD blocks of figure-shaped and Slab Grave individuals. Only IBD blocks longer than 12 cM were considered to minimize false positives. The column of this matrix was permuted for the permutation test. (B) Average IBD sharing within and between groups. The average IBD shared among the four groups were calculated by dividing the total IBD blocks by the number of all possible pairs within and between the groups. (C) IBD Sharing Matrix between figure-shaped Slab Grave individuals. The matrix shows the sum of IBD blocks shared between figure-shaped and Slab Grave individuals, grouped into the four categories. We divided 26 individuals into four groups based on cultural affiliation and geographical location: SlabGrave_center (Sc; n=6), SlabGrave_other (So; n=9), FigureBurial_center (Fc; n=3), and FigureBurial_other (Fo; n=8). IBD sharing was then calculated among these groups. Enrichment of IBD sharing between groups from the same region over time, particularly between SlabGrave_center and FigureBurial_center, would suggest genetic continuity within the region without significant population replacement, and thus support the cultural diffusion model of the Slab Grave expansion. Only IBD blocks longer than 12 cM were considered to minimize false positives. The column of this matrix was permuted for the permutation test. (D) Average IBD shared between four groups. The average IBD shared among the four groups were calculated by dividing the total IBD blocks by the number of all possible pairs within and between the groups. (E) The sum IBD shared between FigureBurial and SlabGrave, based on the geographical location of FigureBurial. The sum of IBD shared between Fe and SlabGrave is compared to the total IBD shared between FigureBurial and SlabGrave.

Supplementary Data 14. f_4-symmetry test among three DSKC subgroups to assess the genetic homogeneity of Mongolia_LBA_DSKC (n=41). To evaluate the genetic homogeneity within the Mongolia_LBA_DSKC group (n=41), we divided the individuals into three subgroups and performed f_4-symmetry tests for all three pairwise comparisons: 1) CentralMongolia_LBA_DSKC (n=14), newly reported individuals from central Mongolia. 2) DSKC1 (n=16), individuals from Khovsgol Aimag previously reported in Jeong et al. (2018). 3) DSKC2 (n=11), a geographically diverse set of DSKC individuals reported in Wang et al. (2021). CentralMongolia_LBA_DSKC and DSKC1 were all processed at the Max Planck Institute and DSKC2 was processed at Harvard University. We computed f_4-statistics of the form f_4(Mbuti.DG, worldwide; X, Y), where X and Y are the DSKC subgroups and the reference panel consists of 300 ancient and present-day populations worldwide. Populations yielding |Z| > 0.05 are highlighted in red. (A) f_4-symmetry tests to compare CentralMongolia_LBA_DSKC and DSKC1. Only 3 of 300 reference populations produced |Z| > 0.05, indicating minimal genetic differentiation. (B) f_4-symmetry test to compare CentralMongolia_LBA_DSKC vs. DSKC2. 37 of 300 populations showed |Z| > 0.05, indicating minimal genetic differentiation. (C) f_4-symmetry tests to compare DSKC1 and DSKC2. 5 of 300 populations showed |Z| > 0.05, indicating minimal genetic differentiation. Together, these results support overall genetic homogeneity within Mongolia_LBA_DSKC, despite differences in geographic origin and laboratory processing.

Supplementary Data 15. QpAdm modeling of Mongolia_LBA_DSKC and Altai_MLBA populations. We conducted the qpAdm analysis of Mongolia_LBA_DSKC and Altai_MLBA. Models with qpAdm p-values >0.05 are highlighted in grey and models presented in the main text are

highlighted in bold. We used a base set of ten outgroups ("Base"): Mbuti, Onge, Natufian, Iran_N, Villabruna, Ami, Mixe, Anatolia_N as a default. We also presented the results with an additional outgroup (Krasnoyarsk_MLBA, Sintashta_MLBA, Afanasievo, and Khemtseg) to show the robustness of new models. (A) Summary of the group-based qpAdm analysis of Mongolia_LBA_DSKC. We performed group-based qpAdm analysis of Mongolia_LBA_DSKC (n=41) and found that previously published admixture models do not fit for the new supergroup. (B) Summary of the group-based qpAdm analysis of Altai_MLBA. We performed group-based qpAdm analysis of Altai_MLBA (n=9) and found that they were well explained by the two-way mixture between Mongolia_LBA_DSKC and the Western Steppe ancestry, consistent with the previous studies. (C) Summary of qpAdm estimates for autosomes and the X chromosome in Mongolia_LBA_DSKC. We conducted qpAdm analyses separately for autosomes and the X chromosome in Mongolia_LBA_DSKC (n=41), revealing a slight male-biased contribution of Baikal_LNBA ancestry. (D) Summary of qpAdm estimates for autosomes and the X chromosome in Altai_MLBA. We conducted qpAdm analyses separately for autosomes and the X chromosome in Altai_MLBA (n=9), revealing a slight male-biased contribution of Western Steppe ancestry.

Supplementary Data 16. QpAdm modeling of subgroups and individuals from Mongolia_LBA_DSKC. We performed qpAdm analysis to demonstrate that a three-way admixture model of Baikal_LNBA + Afanasievo/Khemtseg + ANA provides a good fit for all subgroups and individuals of Mongolia_LBA_DSKC (n = 41). Models with qpAdm p-values > 0.05 are highlighted in grey, and models presented in the main text are highlighted in bold. We used a base set of ten outgroups ("Base"): Mbuti, Onge, Natufian, Iran_N, Villabruna, Ami, Mixe, Anatolia_N as the default. (A) Summary of group-based qpAdm analysis of the subgroups of Mongolia_LBA_DSKC. We conducted group-based qpAdm analysis on three subgroups of Mongolia_LBA_DSKC and found that all are well explained by the same three-way model that fits the entire Mongolia_LBA_DSKC population. (B) Summary of individual-based qpAdm analysis of Mongolia_LBA_DSKC. We performed individual-based qpAdm analysis and found that almost all individuals are also well explained by the same three-way model.

Supplementary Data 17. Summary of IBD sharing pattern of 59 individuals from LBA and EIA Mongolia. We divided 59 individuals into four populations based on their cultural affiliations and genetic profiles: Mongolia_LBA_DSKC (n=25), figure-shaped (n=11), Altai_MLBA (n=8), and SlabGrave (n=15). We calculated the number of pairs sharing IBD blocks, the proportion of the number of pairs sharing IBD blocks, the number of IBD blocks, cumulative length (sum) of IBD blocks, and average length of IBD blocks shared between two populations. Only IBD blocks longer than 12 cM were considered to minimize false positives. (A) The number of pairs of individuals sharing IBD blocks between two populations. (B) The proportion of the number of pairs of individuals sharing IBD blocks between two populations. (C) The Number of IBD blocks shared between two populations. (D) Cumulative length (sum) of IBD blocks (cM) shared between two populations. (E) Average length of IBD blocks (cM) shared between two populations.

Supplementary Data 18. QpAdm modeling of three individuals from central Mongolia. We performed qpWave and qpAdm analysis on three newly sequenced individuals from central Mongolia: KHG005, associated with the Xiongnu empire; SOV001, associated with the Uyghur Empire; and AMY001, associated with the Mongol Empire. Using previously published admixture models for Xiongnu, Uyghur and Mongol individuals, we found that KHG005 is genetically indistinguishable with SlabGrave1 and the other two individuals are explained by the two-way admixture model involving

SlabGrave1 and Sarmatian/Alan. Models with qpWave/qpAdm p-values >0.05 are highlighted in grey. We used a set of eight outgroups: Mbuti, Onge, Natufian, Iran_N, Villabruna, Ami, Mixe, Anatolia_N.
