# Supplementary material for: Slab Grave expansion disrupted long co-existence of distinct Bronze Age herders in central Mongolia
Source: Nat Commun. 2025 Sep 25;16:8420. doi: 10.1038/s41467-025-63789-1 (PMC12462455; doi:10.1038/s41467-025-63789-1)
Supplement: Supplementary file 4 — Reporting Summary [file 41467_2025_63789_MOESM4_ESM.pdf]

nature portfolio

Corresponding author(s): Choongwon Jeong

Last updated by author(s): Aug 5, 2025

Reporting Summary

Nature Portfolio wishes to improve the reproducibility of the work that we publish. This form provides structure for consistency and transparency in reporting. For further information on Nature Portfolio policies, see our Editorial Policies and the Editorial Policy Checklist.

Statistics

For all statistical analyses, confirm that the following items are present in the figure legend, table legend, main text, or Methods section.

n/a	Confirmed	
☐	☒	The exact sample size (*n*) for each experimental group/condition, given as a discrete number and unit of measurement
☐	☒	A statement on whether measurements were taken from distinct samples or whether the same sample was measured repeatedly
☐	☒	The statistical test(s) used AND whether they are one- or two-sided<br>*Only common tests should be described solely by name; describe more complex techniques in the Methods section.*
☒	☐	A description of all covariates tested
☒	☐	A description of any assumptions or corrections, such as tests of normality and adjustment for multiple comparisons
☐	☒	A full description of the statistical parameters including central tendency (e.g. means) or other basic estimates (e.g. regression coefficient) AND variation (e.g. standard deviation) or associated estimates of uncertainty (e.g. confidence intervals)
☐	☒	For null hypothesis testing, the test statistic (e.g. *F*, *t*, *r*) with confidence intervals, effect sizes, degrees of freedom and *P* value noted<br>*Give P values as exact values whenever suitable.*
☒	☐	For Bayesian analysis, information on the choice of priors and Markov chain Monte Carlo settings
☒	☐	For hierarchical and complex designs, identification of the appropriate level for tests and full reporting of outcomes
☒	☐	Estimates of effect sizes (e.g. Cohen's *d*, Pearson's *r*), indicating how they were calculated

Our web collection on statistics for biologists contains articles on many of the points above.

Software and code

Policy information about availability of computer code

Data collection	Illumina sequence data were processed with the following programs to generate the genotype data used in the analysis: AdapterRemoval v2.3.1, BWA v0.7.17, DeDeup v0.12.8, samtools v1.19.2, pileupCaller in sequenceTools v1.5.2 (https://github.com/stschiff/sequenceTools), mapDamage v2.2.1, ANGSD v0.941, Schmutzi v1.5.7. These programs are publicly available. All scripts and code used in the analysis are publicly available at https://zenodo.org/records/16743201.
Data analysis	Population genetic data analysis in this study was performed using the following publicly available programs: HaploGrep v2.1.20, yHaplo (https://github.com/alexhbnr/yhaplo), KIN v3.1.2, hapROH (https://github.com/hringbauer/hapROH), smartpca v18140, qpDstat v980, qpWave v1520, qpAdm v1520, ancIBD v0.7, bcftools v1.19, GLIMPSE1, Ped-sim v1.4, R v4.3.1 with a publicly available library stats v4.3.1. Non-default parameters used in our analysis are described in the Methods section. All scripts and code used in the analysis are publicly available at https://zenodo.org/records/16743201.<br>The base map in Figure 1A is in the public domain and accessible through the Natural Earth website (https://www.naturalearthdata.com/downloads/10m-raster-data/). The base map in Figure 1B is based on Google Earth Pro, version 7.3.6 (image © Maxar Technologies, © 2024 Airbus, ©2024 CNES/Airbus). The base maps in Supplementary Figures 10 and 12 were created in R v4.3.1 using a publicly available library maps v3.4.0.

For manuscripts utilizing custom algorithms or software that are central to the research but not yet described in published literature, software must be made available to editors and reviewers. We strongly encourage code deposition in a community repository (e.g. GitHub). See the Nature Portfolio guidelines for submitting code & software for further information.

Data

Policy information about availability of data

All manuscripts must include a data availability statement. This statement should provide the following information, where applicable:

- Accession codes, unique identifiers, or web links for publicly available datasets
- A description of any restrictions on data availability
- For clinical datasets or third party data, please ensure that the statement adheres to our policy

All data needed to evaluate the conclusions in the paper are present in the paper and/or the Supplementary Materials. All newly generated sequencing data reported in this study, including raw reads (FASTQ) and aligned reads (BAM), are available from the European Nucleotide Archive under the accession number PRJEB83289 [https://www.ebi.ac.uk/ena/browser/view/PRJEB83289]. Previously published datasets analyzed in this study are available from the European Nucleotide Archive, with individual accession numbers provided in Supplementary Data 2. The 1240K panel genotype data for the newly generated individuals are available in the Edmond Data Repository of the Max Planck Society at [https://edmond.mpg.de/dataset.xhtml?persistentId=doi:10.17617/3.6OUL8B] and on Zenodo at https://zenodo.org/records/16743201. The base map in Figure 1A is in the public domain and accessible through the Natural Earth website (https://www.naturalearthdata.com/downloads/10m-raster-data/). The base map in Figure 1B is based on Google Earth Pro, version 7.3.6 (image © Maxar Technologies, © 2024 Airbus, ©2024 CNES/Airbus). The base maps in Supplementary Figures 10 and 12 were created in R v4.3.1 using a publicly available library maps v3.4.0. Source data for Figures 1, 3, and 4, and Supplementary Figs. 9, 10, 13, and 15 are available in the Supplementary Data file. Source data for Figures 1, 2, and 3, and Supplementary Figs. 3, 4, 8, 11, 12, 14, and 16 are available at https://zenodo.org/records/16743201. The archaeological human remains studied in this study are being housed in and managed by the Institute of Archaeology, Mongolian Academy of Sciences, Mongolia.

Research involving human participants, their data, or biological material

Policy information about studies with human participants or human data. See also policy information about sex, gender (identity/presentation), and sexual orientation and race, ethnicity and racism.

Reporting on sex and gender	*Use the terms sex (biological attribute) and gender (shaped by social and cultural circumstances) carefully in order to avoid confusing both terms. Indicate if findings apply to only one sex or gender; describe whether sex and gender were considered in study design; whether sex and/or gender was determined based on self-reporting or assigned and methods used.* *Provide in the source data disaggregated sex and gender data, where this information has been collected, and if consent has been obtained for sharing of individual-level data; provide overall numbers in this Reporting Summary. Please state if this information has not been collected.* *Report sex- and gender-based analyses where performed, justify reasons for lack of sex- and gender-based analysis.*
Reporting on race, ethnicity, or other socially relevant groupings	*Please specify the socially constructed or socially relevant categorization variable(s) used in your manuscript and explain why they were used. Please note that such variables should not be used as proxies for other socially constructed/relevant variables (for example, race or ethnicity should not be used as a proxy for socioeconomic status).* *Provide clear definitions of the relevant terms used, how they were provided (by the participants/respondents, the researchers, or third parties), and the method(s) used to classify people into the different categories (e.g. self-report, census or administrative data, social media data, etc.)* *Please provide details about how you controlled for confounding variables in your analyses.*
Population characteristics	*Describe the covariate-relevant population characteristics of the human research participants (e.g. age, genotypic information, past and current diagnosis and treatment categories). If you filled out the behavioural & social sciences study design questions and have nothing to add here, write "See above."*
Recruitment	*Describe how participants were recruited. Outline any potential self-selection bias or other biases that may be present and how these are likely to impact results.*
Ethics oversight	*Identify the organization(s) that approved the study protocol.*

Note that full information on the approval of the study protocol must also be provided in the manuscript.

Field-specific reporting

Please select the one below that is the best fit for your research. If you are not sure, read the appropriate sections before making your selection.

☐ Life sciences ☐ Behavioural & social sciences ☒ Ecological, evolutionary & environmental sciences

For a reference copy of the document with all sections, see nature.com/documents/nr-reporting-summary-flat.pdf

Ecological, evolutionary & environmental sciences study design

All studies must disclose on these points even when the disclosure is negative.

Study description	This study includes whole genome or genome-wide sequencing of 30 ancient individuals from central Mongolia, spanning the Late Bronze Age, Early Iron Age, and subsequent periods, dated between 1500 BCE and 1400 CE. Sequencing coverage ranges 0.019-9.035x. Ancient genomes come from six sites in two river valleys: Ar Bulan (n=1), Ar Modny Adag (n=1), Maikhan Tolgoi (n=15) and OOR-284 (n=1) from the Upper Orkhon Valley, and Khuruugiin uzuur (n=7) and Tsats Tolgoi (n=5) from the Upper Tamir Valley.

Research sample	The research samples newly generated in this study comprises 30 ancient human genomes from multiple archaeological sites in central Mongolia. These individuals were primarily selected to represent key archaeological cultures of the Late Bronze Age—namely, the figure-shaped and Deer Stone-Khirgisuur Complex (DSKC) cultures—and the Early Iron Age Slab Grave culture, as well as to incorporate available individuals from subsequent periods, including the Xiongnu, Uyghur, and Mongol. Based on chronology, cultural affiliation, and genetic profiles, we grouped them into seven analytical units: CentralMongolia_LBA_DSKC (Late Bronze Age individuals associated with the DSKC; n=14), CentralMongolia_LBA_DSKC_outlier (DSKC-associated individuals with slightly different genetic profiles from the other DSKC individuals; n=2), CentralMongolia_LBA_FigureBurial (individuals from figure-shaped burials; n=3), CentralMongolia_EIA_SlabGrave (Early Iron Age Slab Grave individuals; n=8), Xiongnu (n=1), Uyghur (n=1), and Mongol (n=1). For Identity-By-Descent-based analyses, individuals sharing the same cultural affiliation were grouped into a single analytical unit. Published ancient genomes from neighboring regions or with genetic or cultural affinities to the newly sequenced individuals were also analyzed and are available in the European Nucleotide Archive (Supplementary Data 2).
Sampling strategy	No sample-size selection was performed prior to the study. To produce ancient genomes reported in this study, we screened 32 accessible skeletal elements from the relevant geographic regions and time periods, and produced in-depth sequencing data for 30 samples with sufficient endogenous DNA preservation and without substantial contamination. All exclusions and criteria are detailed in the Methods section.
Data collection	DNA extraction and library preparation for 32 skeletal samples were conducted at the Max Planck Institute for Evolutionary Anthropology (MPI-EVA) by Raphaela Stahl and Lena Semerau. Shallow sequencing was performed on an Illumina HiSeq 4000 using 1x75 bp chemistry to assess human DNA preservation. Of these, 30 samples were selected for the in-solution DNA capture targeting 1,233,013 ancestry-informative single-nucleotide polymorphisms. Target enrichment was carried out at MPI-EVA by Raphaela Stahl and Lena Semerau, and the enriched libraries were sequenced at MPI-EVA on an Illumina HiSeq 4000 using 1x75 bp chemistry and at the Bauer Core Facility of Harvard University on an Illumina NovaSeq 6000 S4 flow cell using 2x100 bp chemistry. Additionally, 10 samples were whole-genome sequenced at the Bauer Core Facility of Harvard University on an Illumina NovaSeq 6000 S4 flow cell using 2x100 bp chemistry.
Timing and spatial scale	Laboratory works and sequencing were conducted over the period from June 2020 to August 2023. Samples were taken from various archaeological sites in central Mongolia. Detailed information of the archaeological samples newly generated in this study are provided in Figure 1 and Supplementary Data 1.
Data exclusions	We excluded 2 out of the 32 initially screened samples due to low levels of endogenous human DNA, which prohibits genome-scale sequencing. The remaining samples were all included in this study as they exhibited low contamination levels and no close genetic relationships (first-degree genetic relationships). All exclusions and criteria are detailed in the Methods section.
Reproducibility	We took multiple individuals from each archaeological site, if available, to support the representativeness of their genetic profiles. All experimental and analytical procedures yielded consistent results across replicates. Each capture library was sequenced multiple times, and 10 out of 30 individuals with sufficient endogenous DNA content were additionally sequenced using shotgun libraries; all library preparation and sequencing approaches yielded consistent and high-quality results across individuals. Population genetic analyses, including qpAdm, qpWave, and IBD-based methods, produced consistent outcomes across independent runs. To ensure transparency and reproducibility, all custom scripts and parameter settings used in the analyses are available at Zenodo (https://zenodo.org/records/16743201).
Randomization	Ancient genomes were first analyzed by each individual, and then were allocated into the analysis group based on their absolute date (14C dating), cultural affiliation, and their individual genetic profile. Randomization is not applicable because this study is observational and includes no treatment nor case/control comparison.
Blinding	There was no experimental treatment of samples involved in this study that requires blinding. Data analysis was performed based on the analysis groups that were defined by external information (archaeological context and date).

Did the study involve field work? ☐ Yes ☒ No

Reporting for specific materials, systems and methods

We require information from authors about some types of materials, experimental systems and methods used in many studies. Here, indicate whether each material, system or method listed is relevant to your study. If you are not sure if a list item applies to your research, read the appropriate section before selecting a response.

Materials & experimental systems

n/a	Involved in the study
☒	☐ Antibodies
☒	☐ Eukaryotic cell lines
☐	☒ Palaeontology and archaeology
☒	☐ Animals and other organisms
☒	☐ Clinical data
☒	☐ Dual use research of concern
☒	☐ Plants

Methods

n/a	Involved in the study
☒	☐ ChIP-seq
☒	☐ Flow cytometry
☒	☐ MRI-based neuroimaging

Palaeontology and Archaeology

Specimen provenance	The archaeological human remains from the Upper Orkhon Valley were excavated between 2009 and 2022 under cooperation agreements between the Mongolian Academy of Sciences, Institute of Archaeology, and the Institute of Pre- and Early Historical Archaeology, University of Bonn, Germany. These remains were exported to MPI-EVA on 12 March 2019 for scientific investigation under license agreement A0128802, granted by the Mongolian National Chamber of Commerce and Industry. The excavations in the Upper Tamir Valley were executed by the Mongolian Academy of Sciences, Institute of Archaeology, and the Musée d'Anthropologie préhistorique de Monaco. The human remains from these excavations were exported to MPI-EVA on 23 October 2018 under license agreement A0125623, granted by the Mongolian National Chamber of Commerce and Industry.
Specimen deposition	The archaeological human remains studied in this manuscript are housed and managed by the Institute of Archaeology, Mongolian Academy of Sciences, and are accessible using the archaeological IDs provided in Supplementary Data 1 upon request to the institute and the original excavators.
Dating methods	No new dates are provided in this manuscript. All raw and calibrated dates referenced in this manuscript were previously reported in reference 14 (with numbering consistent with the main text).

☒ Tick this box to confirm that the raw and calibrated dates are available in the paper or in Supplementary Information.

Ethics oversight	This study is based on previously excavated archaeological remains and included no new excavation effort nor study of live human or animal subjects. Therefore the study protocols used in this study are not the subject of approval by IRB/IACUC. The access to the remains was approved by the Mongolian National Chamber of Commerce and Industry, as part of the permitting and export process (see above).

Note that full information on the approval of the study protocol must also be provided in the manuscript.

Plants

Seed stocks	*Report on the source of all seed stocks or other plant material used. If applicable, state the seed stock centre and catalogue number. If plant specimens were collected from the field, describe the collection location, date and sampling procedures.*
Novel plant genotypes	*Describe the methods by which all novel plant genotypes were produced. This includes those generated by transgenic approaches, gene editing, chemical/radiation-based mutagenesis and hybridization. For transgenic lines, describe the transformation method, the number of independent lines analyzed and the generation upon which experiments were performed. For gene-edited lines, describe the editor used, the endogenous sequence targeted for editing, the targeting guide RNA sequence (if applicable) and how the editor was applied.*
Authentication	*Describe any authentication procedures for each seed stock used or novel genotype generated. Describe any experiments used to assess the effect of a mutation and, where applicable, how potential secondary effects (e.g. second site T-DNA insertions, mosiacism, off-target gene editing) were examined.*
